# Isotopic compositions of ground ice in near-surface permafrost in relation to vegetation and microtopography at the Taiga–Tundra boundary in the Indigirka River lowlands, northeastern Siberia

**DOI:** 10.1371/journal.pone.0223720

**Published:** 2019-10-10

**Authors:** Shinya Takano, Atsuko Sugimoto, Shunsuke Tei, Maochang Liang, Ryo Shingubara, Tomoki Morozumi, Trofim C. Maximov

**Affiliations:** 1 Graduate School of Environmental Science, Hokkaido University, Sapporo, Japan; 2 Faculty of Environmental Earth Science, Hokkaido University, Sapporo, Japan; 3 Arctic Research Center, Hokkaido University, Sapporo, Japan; 4 Global Station for Arctic Research, Hokkaido University, Sapporo, Japan; 5 North-Eastern Federal University in Yakutsk, Yakutsk, Sakha, Russia; 6 Institute for Biological Problems of Cryolithozone, Siberian Branch of the Russian Academy of Sciences, Yakutsk, Sakha, Russia; Oakland University, UNITED STATES

## Abstract

The warming trend in the Arctic region is expected to cause drastic changes including permafrost degradation and vegetation shifts. We investigated the spatial distribution of ice content and stable isotopic compositions of water in near-surface permafrost down to a depth of 1 m in the Indigirka River lowlands of northeastern Siberia to examine how the permafrost conditions control vegetation and microtopography in the Taiga–Tundra boundary ecosystem. The gravimetric water content (GWC) in the frozen soil layer was significantly higher at microtopographically high elevations with growing larch trees (i.e., tree mounds) than at low elevations with wetland vegetation (i.e., wet areas). The observed ground ice (ice-rich layer) with a high GWC in the tree mounds suggests that the relatively elevated microtopography of the land surface, which was formed by frost heave, strongly affects the survival of larch trees. The isotopic composition of the ground ice indicated that equilibrium isotopic fractionation occurred during ice segregation at the tree mounds, which implies that the ice formed with sufficient time for the migration of unfrozen soil water to the freezing front. In contrast, the isotopic data for the wet areas indicated that rapid freezing occurred under relatively non-equilibrium conditions, implying that there was insufficient time for ice segregation to occur. The freezing rate of the tree mounds was slower than that of the wet areas due to the difference of such as soil moisture and snow cover depends on vegetation and microtopography. These results indicate that future changes in snow cover, soil moisture, and organic layer, which control underground thermal conductivity, will have significant impacts on the freezing environment of the ground ice at the Taiga–Tundra boundary in northeastern Siberia. Such changes in the freezing environment will then affect vegetation due to changes in the microtopography of the ground surface.

## Introduction

Permafrost zones occupy approximately 24% (22.79 million km^2^) of the land area in the Northern Hemisphere [1]. Taiga and Tundra ecosystems are located on the permafrost and play an important role in the global carbon cycle as carbon sinks and/or CH_4_ sources [2]. The warming trend in high-latitude areas caused by Arctic amplification (e.g., [3]) is expected to lead to drastic changes such as permafrost degradation (e.g., [4]), forest productivity changes (e.g., [5, 6]), and tree line shifts (e.g., [7, 8]). Therefore, it is necessary to understand how permafrost and vegetation respond to climate change in high-latitude areas.

The Taiga–Tundra boundary covers over 1.9 million km^2^ of the circum-Arctic region [9] and is expanding because trees and shrubs are currently increasing in tundra ecosystems [8, 10]. In addition, thermokarst processes in the permafrost change the vegetation from forest to wetland at this boundary (e.g., [11]), which may cause CH_4_ emissions.

Ground ice is part of the permafrost and can control the vegetation structure and composition. In the Arctic tundra, polygon mires are a typical microtopographic landscape [12] and cover approximately 250,000 km^2^ of the circum-Arctic region [13]. They are formed by the growth of ground ice such as ice wedges [14], and a clear zonation of vegetation has been observed corresponding to the polygonal pattern in the microtopography [15–17]. This means that ground ice forms a ridge (i.e., relatively high ground surface and high frost table) that causes relatively dry soil conditions [18–20]; thus, it controls the vegetation structure and composition in the Arctic tundra area. Although polygon mires are mainly covered by wetland vegetation, the microtopographic landscape may also affect the vegetation structure and composition in the Taiga–Tundra boundary ecosystem. It has been reported that near-surface ground ice characteristics are closely related to vegetation type owing to physical conditions associated with differences in the alluvial environments of Arctic Canada [21], and there are many other studies on ground ice content in relation to vegetation and microtopography (e.g., [22–25]).

The ground ice (e.g., ice wedges and segregation ice [26]) in permafrost is generally formed by the infiltration of water, which is kept in the soil and freezes. Ice segregation frequently occurs in the transition zone, which is at the uppermost layer of the permafrost and alternates between an active layer and permafrost over periods ranging from less than a decade to multiple centuries [27, 28]. The transition zone experiences favorable thermal and hydrological conditions for ice segregation, such as water saturation and slow freezing, and frequently forms ice lenses parallel to the ground surface with high ice content.

The freezing rate is one of the most important factors for ground ice formation in soil. Slow freezing can provide sufficient time for unfrozen soil water to slowly migrate to the freezing front. The freezing rate depends on the thermal conductivity in the surface soil, which fluctuates due to such as soil moisture, soil type, snow cover, and vegetation [29−31]. The thermal conductivity (i.e., the freezing rate) increases when higher soil moisture, less organic layer, and less snow cover, and inversely decreases when lower soil moisture, more organic layer, and more snow cover. Therefore, the freezing rate can be affected by these factors.

Stable isotope ratios of water are a useful tool (e.g., [32, 33]) for determining source water and have long been utilized in studies on the water cycle (e.g., [34–36]). The isotopic compositions were expressed in delta notation relative to Vienna Standard Mean Ocean Water (VSMOW). The delta values were defined as follows:

$$\delta^{18}O\;or\;\delta D=\left\{\left(R_{sample}-R_{VSMOW}\right)/R_{VSMOW}\right\}\times 1000\;(‰),$$ (1)

where R_sample_ and R_VSMOW_ are the water isotopic ratios (^18^O/^16^O or D/H) of the samples and VSMOW, respectively. Deuterium excess (d-excess), which is an indicator of non-equilibrium processes (e.g., [36, 37]), was calculated from δ^18^O and δD as follows [35]:

$$d‐\mathrm{excess}=\delta D‐8\times \delta^{18}O.$$ (2)

The d-excess and the regression slope between δ^18^O and δD can be used as an indicator for the equilibrium or non-equilibrium state during such as the evaporation process (e.g., [36, 37]) and the freezing process [38–40]. The water isotope ratios of the ice provide some information about the freezing environment during ground ice formation (e.g., [38, 39, 41]), as has been observed in northern Siberia [42–52] and other Arctic and sub-Arctic regions [53–59]. The δ^18^O values of the ice have been reported to be enriched by 2.8–3.1‰ compared to the source water when frozen under isotopically equilibrium conditions [38, 40], and the delta values gradual decreased from the first formed ice to the subsequent ices according to Rayleigh-type isotope fractionation (S1 Appendix). In the δD−δ^18^O plot, the slope of the regression line of the isotopic compositions of ice formed during the equilibrium freezing shows from approximately 6 to 7.3, and that formed under the non-equilibrium freezing shows lower values [38, 39].

Iwahana et al. [50] observed the current thermal regime of the active layer and spatial variations in the ice content, soil mechanical characteristics, cryostratigraphy, and water stable isotope ratio of near-surface permafrost in the Indigirka River lowlands of northeastern Siberia. They found similarities in the geocryological characteristics in the research area, which suggests that the formation of the upper 1 m cryostratigraphy may have been affected by similar hydrological conditions in the area.

For better understanding of the possible responses of the ground ice, microtopography, and vegetation structure and composition in the Arctic Taiga–Tundra boundary to future climate change, it is necessary to assess the ground ice formation conditions and how ground ice is related to the vegetation and microtopography. Therefore, we examined the ice content and isotopic compositions of near-surface permafrost down to a depth of 1 m at the Taiga–Tundra boundary near Chokurdakh in the Indigirka River lowlands of northeastern Siberia.

## Materials and methods

### Study sites

**Fig 1:**
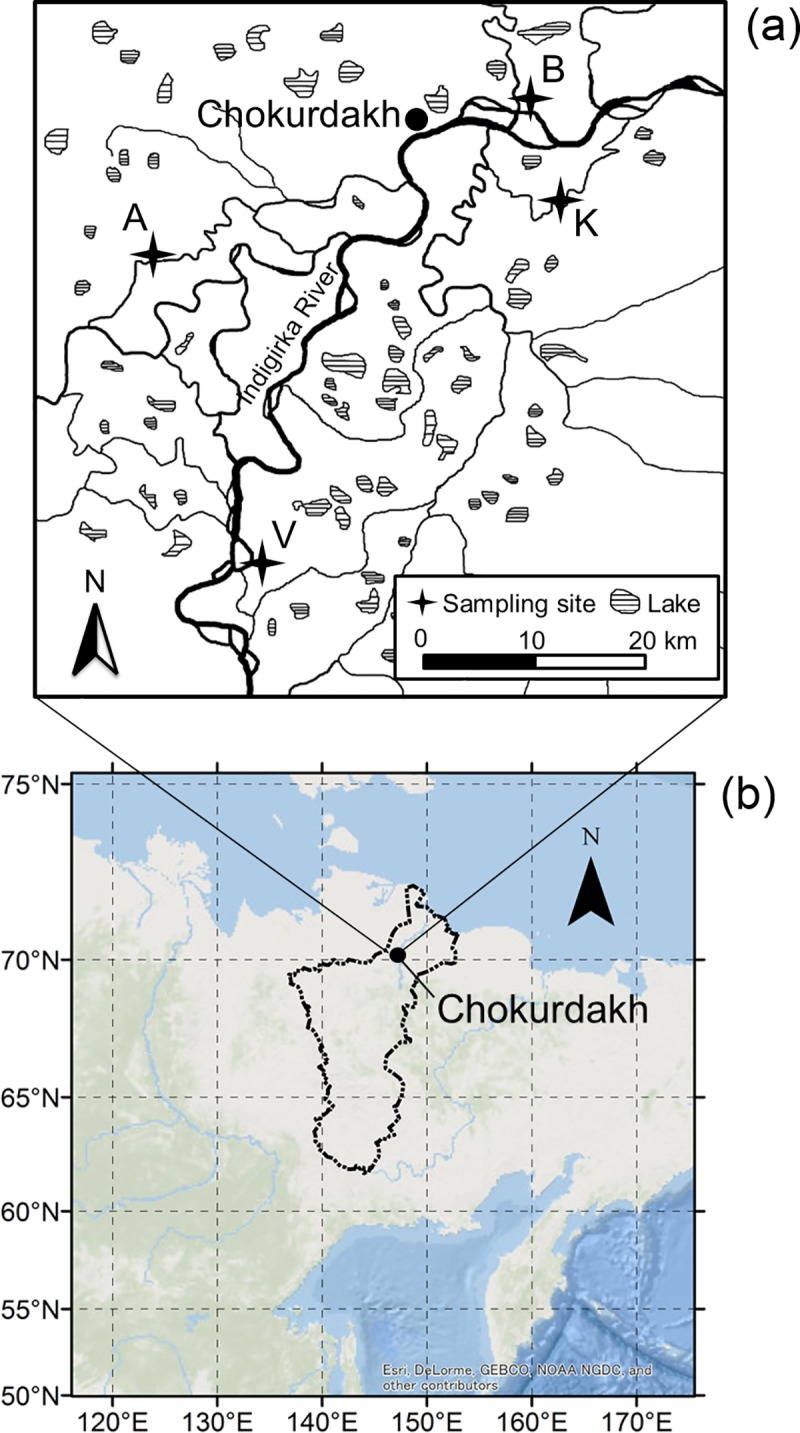
Locations of the study sites in northeastern Siberia. (a) Thick and thin lines represent the Indigirka River and its tributaries, respectively, and the filled areas are lakes. Chokurdakh village and other observation sites V, A, K, and B are also indicated. Republished from Liang et al. [64] under a CC BY license, with permission from Polar Science, original copyright 2014. (b) The Indigirka River basin is shown with a line. Republished from Ocean Basemap (https://services.arcgisonline.com/ArcGIS/rest/services/Ocean_Basemap/MapServer) under a CC BY license, with permission from ESRI, original copyright 2018.

We selected four observation sites with different densities of larch trees (from least to highest): Boydom (site B), Kodac (site K), Allikha (site A) and Verkhniy Khatistakh (site V). These sites are near Chokurdakh (70°37′N, 147°53′E), which is downstream of the Indigirka River in northeastern Siberia (Fig 1 and Table 1). All field studies were organized by the Institute for Biological Problems of Cryolithozone, Siberian Branch of the Russian Academy of Sciences, Yakutsk, Sakha, Russia with permissions from Federal Service for Technical and Export Control. The mean annual air temperature is −13.9°C, and the mean monthly air temperatures in January and July are −34.2 and 10.0°C, respectively. The annual mean precipitation at Chokurdakh from 1950–2008 was 210 mm according to the Baseline Meteorological Data in Siberia (BMDS) Version 5.0 [60]. Summer temperatures during the past 50 years have shown a warming trend, winter precipitation during the past century showed an increase trend [61, 62]. In recent years, larch and tall shrub coverage has increased [8], and an increase in invasive (expanded) plant species from more southerly regions has been reported [63].

**Table 1:** Summary of the observation and sampling data.

Site	Latitude Longitude	Sampling point	Sampling date	Sampling depth (cm)	Thaw depth (cm)	Landscape & vegetation category	Profile pattern
B	70゜38'15"N 148゜09'17"E	B1	9 July 2011	90	20	Tree mound	i
		B2	9 July 2011	90	30	Wet area	i
		B3	20 July 2012	103	26	Tree mound	i
		B4	20 July 2012	92	40	Wet area	i
K	70゜33'48"N 148゜15'51"E	K1	10 July 2011	90	20	Tree mound	i, ii
		K2	10 July 2011	90	20	Tree mound	i, iii
		K3	10 July 2011	90	30	Wet area	iv
		K4	11 July 2011	90	20	Tree mound	i, iii
		K5	11 July 2011	90	20	Tree mound	i
		K6	11 July 2011	90	20	Intermediate	i
		K7	16 July 2012	100	50	Tree mound	ii
		K8	17 July 2012	98	13	Tree mound	ii
		K9	19 July 2012	99	20	Tree mound	ii
		K10	19 July 2012	97	30	Wet area	iv
		K11	19 July 2012	104	33	Wet area	iv
		K12	20 July 2012	102	30	Intermediate	iv
V	70゜15'07"N 147゜28'08"E	V1	23 July 2011	90	20	Tree mound	i
		V2	23 July 2011	90	30	Wet area	i
		V3	26 July 2012	98	42	Tree mound	anomaly
		V4	26 July 2012	94	40	Wet area	iv
A	70゜30'56"N 147゜18'15"E	A1	24 July 2011	90	40	The slope of a hill	i
		A2	24 July 2011	90	20	Intermediate	iv

This observation area is underlain by continuous permafrost. Normally, snowmelt and active layer thawing start in the late May through to early June; the growing season is from late June to early August. The average thaw depth observed between 3 July and 9 August at the study sites was 31±12 cm [65], whereas the maximum thaw depth was found at the end of thaw season (the first half of September). The active layer begins to freeze in the second half of September through to October and freezes completely between November and December.

The observation area is located on the Taiga–Tundra boundary. This area has mound-shaped landforms and wetlands distributed in patches. These mounds look like palsa or peat plateaus and were named “tree mounds” in this study. The vegetation on tree mounds is mainly larches (*Larix cajanderi*, syn. *L*. *gmelinii*), shrubs (including *Betula nana* and *Salix spp*.) and green-mosses (including *Tomentypnum nitens*, *Hylocomium splendens*, and *Aulacomnium turgidum*). Wetlands were labeled as “wet areas” in this study; their vegetation includes sedges (*Eriophorum spp*. and *Carex spp*.) and sphagnum-mosses (including *Sphagnum balticum*, *S*. *squarrosum*, *S*. *angustifolium*). The transitional area between tree mounds and wet areas contained shrubs with sphagnum-mosses and was labeled as “intermediate areas.” Detailed data were obtained on the vegetation, which as classified into several classes (including tree, shrub, willow, cotton-sedge, and *Sphagnum*) based on plant species identified by Morozumi et al. [66]. In this study, the tree, shrub, and willow classes were grouped into tree mounds, and the cotton-sedge and *Sphagnum* classes were grouped into wet areas.

**Fig 2:**
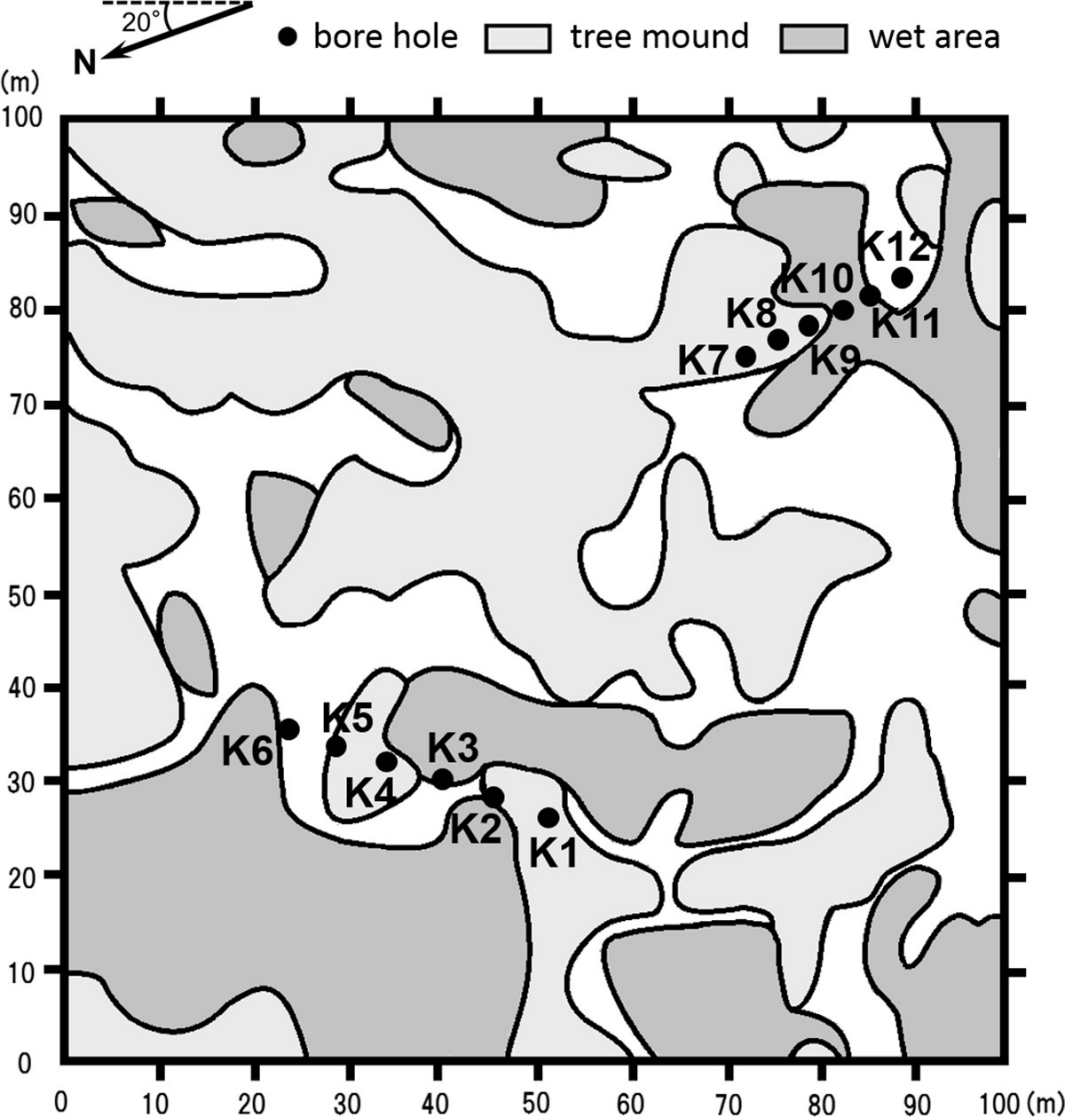
Map of the observation plot at site K. Landscape/vegetation is shown as patterns. Light, thick shaded, and open patterns indicate tree mounds, wet areas, and intermediate areas, respectively. Closed circles represent boreholes from which the frozen soil cores were obtained. Republished from Liang et al. [64] under a CC BY license, with permission from Polar Science, original copyright 2014.

Among the four sites, site B was close to a tundra landscape with several larch trees on an isolated tree mound. Site K (Fig 2) was typical Taiga–Tundra boundary ecosystem. Larch trees grew on tree mounds, while many dead larches were observed in wet areas. Site A was at the foot of a hill with a peak of approximately 40–60 m above the river level. Larch trees grew on the slope of the hill, and steppe tundra covered the top with few or no larch trees. Site V was a relatively dense larch forest in a narrow area along the mainstream of the Indigirka River. Riparian areas were covered by willows and alders. The vegetation at these sites was also described by Liang et al. [64] and Fan et al. [67].

### Observations and samplings

Observation (vegetation structure and composition and microtopography) and sampling (soil and soil water) data were collected at each site, especially for site K, in the periods of 9–24 July 2011 and 16–26 July 2012. The data are summarized in Table 1.

#### Transects and soil sampling

Frozen soil cores down to a depth of approximately 1 m were obtained at all sites with a boring machine (power auger; Tanaka TIA-350S, Nikko Tanaka Engineering Co., Ltd., Chiba, Japan). Core samples with a diameter of approximately 5.5 cm were sampled at intervals of less than 10 cm. Approximately 1 mm was carefully removed from the surface of each sample in order to avoid contamination. Then, each sample was put into a plastic bag. Soil samples of the thaw layer were obtained with a hand shovel and stored similarly as the frozen samples. The organic layer depth and thaw depth were recorded at the same time. At site K, a 30 m transect was set northeast across microtopography and vegetation types and sampled at intervals of 6 m at six points (Points K1–K6 in Fig 2) in 2011. Similarly, a 15 m transect was set south and sampled at intervals of 3 m at six points (Points K7–K12 in Fig 2) in 2012.

Characteristics of soil grain size distribution in the near-surface ground at the study sites were reported by Iwahana et al. [50]. At site K, soils gradually changed from clay loam to silty clay loam downward. Soils showed small variations in loam or silt loam with depth at site B. Soils at site V varied between loam and clay loam with the largest proportion of sand among the sites. Spatially there was no significant difference in soil texture.

#### Vegetation and microtopography

Leveling was conducted along the transect sampling points in the observation plot at site K (Fig 2) with leveling equipment (automatic level; TOPCON AT-B4, Topcon Corporation, Tokyo, Japan) to determine the relative elevations (microtopography) based on the lowest point (0 cm) along the transects. The vegetation composition and landscape were investigated at each sampling point and categorized into three types: tree mounds, wet areas, and intermediate areas (Table 1).

#### Water extraction and measurements of ice/water content

Water in the soil samples of the thaw layer was taken for isotope analysis and stored in glass vials. Soon after thawing of the frozen core samples in the plastic bags, the water (ice) in the samples was taken and stored similarly. The water was directly taken as a supernatant when the ice/water content of the soil samples was high enough. When the ice/water content was low, water was extracted with a centrifuge.

The weight of the soil samples was measured before and after drying to determine the gravimetric water or ice content. The gravimetric water content (GWC) was calculated as follows [26]:

$$GWC\;(\%)=\left(\frac{Weight\;of\;water\;and\;ice\;in\;the\;sample\;(g)}{Dry\;weight\;of\;the\;sample\;(g)}\right)\times 100.$$ (3)

#### River water, precipitation, and snow cover sampling

For isotope analysis, river water from the Indigirka mainstream and tributaries was sampled at 14–30 July 2011 and 25 June–11 August 2012. Precipitation was sampled at 29 June–5 August 2012 during the summer. Because of missing observation data for the precipitation during the 2011 summer sampling period, we also sampled precipitation at 23 June–23 July 2013 and 2–19 July 2014 to identify the approximate water isotopic values of summer precipitation. Snow cover was obtained at 23–26 April 2014 and 15–17 April 2015. The bulk snow was obtained as a core with a diameter of 10 cm and put into a plastic bag to melt. The water of the river, precipitation, and snow cover was kept in glass vials.

### Water isotope analysis

The water samples were analyzed for the stable isotopic composition of water (oxygen and hydrogen) with the CO_2_/H_2_/H_2_O equilibration method using a mass spectrometer (MAT 253, Thermo Fisher Scientific, USA, manufactured in Germany) attached to a Gas Bench (Thermo Fisher Scientific, USA, manufactured in Germany) at the Graduate School of Environmental Science, Hokkaido University, Japan. The precision of the analysis was within ±0.2‰ for δ^18^O and ±2‰ for δD.

In this research, we used the observed δ^18^O and δD values and d-excess. We also used the slope of δD–δ^18^O plot to understand the equilibrium and non-equilibrium fractionations [39, 68]. During the freezing process, when equilibrium fractionation occurs, the slope of δD–δ^18^O plot shows from approximately 6 to 7.3 at 0°C (cf. S1 Appendix). Non-equilibrium fractionation shows lower slope of δD–δ^18^O plot, and when no fractionation occurs, isotopic composition of formed ice is the same composition as water δ^18^O and δD. An intercept of δD–δ^18^O plot is different from d-excess. We did not use the intercept of δD–δ^18^O plot in this research, and the d-excess value was defined as described in the Eq (2).

### Statistical analysis

Mann-Whitney U-tests were conducted to examine differences in the values between the tree mounds and wet areas.

## Results

### Characteristics of permafrost with different vegetation

**Fig 3:**
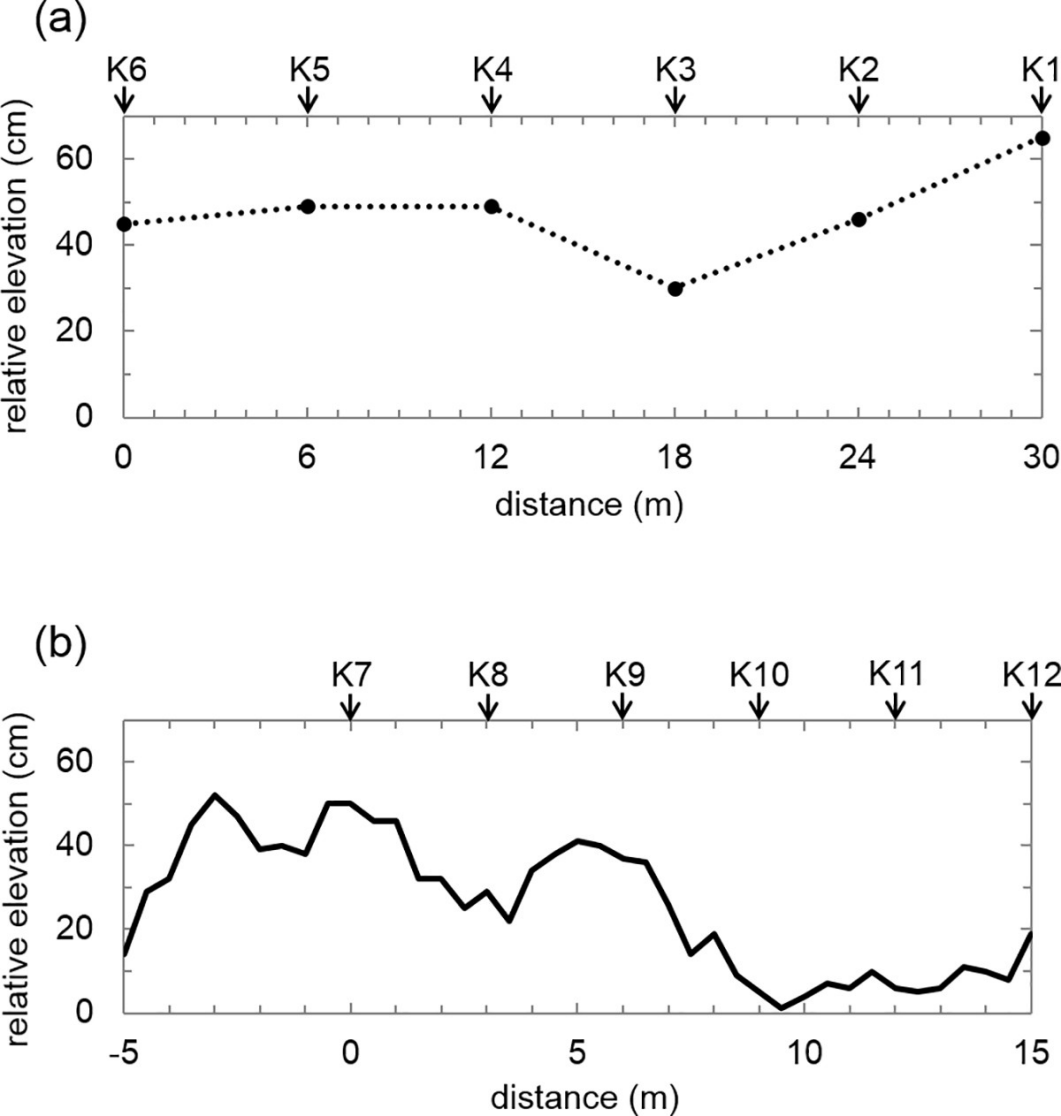
Relative elevations of the transect sampling points in Fig 2. (a) K1–K6 and (b) K7–K12. The relative elevations were determined based on the lowest point (0 cm) in the transects. Because the relative elevations were measured only at the boreholes in (a), the dotted line does not show the microtopography between boreholes.

Fig 3 shows the relative elevations (microtopography) of each sampling point along the two transects at site K. The difference between the lowest and highest points in this sampling area was approximately 50 cm. The averaged relative elevations of the tree mounds and wet areas in site K were 46.4±11.3 cm and 13.7±14.2 cm (Table 2), respectively. Thus, the tree mounds had a higher elevation than the wet areas (p < 0.05).

**Table 2:** Summary of geological and isotopic parameters at the tree mounds and wet areas.

	All sites			Site K		
	tree mound	wet area	significance	tree mound	wet area	significance
Thaw depth (cm)	19.9 ±3.3	30.3 ±6.7	***	18.8 ±2.9	28.6 ±5.0	**
Depth of organic layer (cm)	31.6 ±24.0	26.9 ±8.6	none	36.4 ±27.2	30.8 ±9.6	none
Gravimetric water content of frozen layer (%)	167.3 ±71.1	92.6 ±20.0	***	206.3 ±58.9	100.6 ±19.5	***
δ^18^O of frozen layer (‰)	-21.9 ±0.8	-21.6 ±0.3	none	-21.9 ±1.0	-21.5 ±0.2	none
δ^18^O of thaw layer (‰)	-20.3 ±1.6	-19.9 ±1.4	none	-20.9 ±1.2	-20.9 ±0.9	none
Δδ^18^O of frozen layer (‰)	2.9 ±0.9	2.0 ±1.1	**	2.7 ±0.7	1.5 ±0.3	***
d-excess of frozen layer (‰)	8.1 ±1.9	5.4 ±3.4	*	7.2 ±1.8	3.6 ±2.5	**
d-excess of thaw layer (‰)	10.0 ±2.3	9.1 ±1.3	none	9.3 ±2.4	9.2 ±1.3	none
Relative elevation (cm)				46.4 ±11.3	21.0 ±16.9	**

The average values with the standard deviation (SD) were obtained for all sites and at site K separately. The statistical significance of the differences between the tree mounds and wet areas were as follows:

* = p < 0.1

** = p < 0.05

*** = p < 0.01

none = no difference at the 90% significant level.

**Fig 4:**
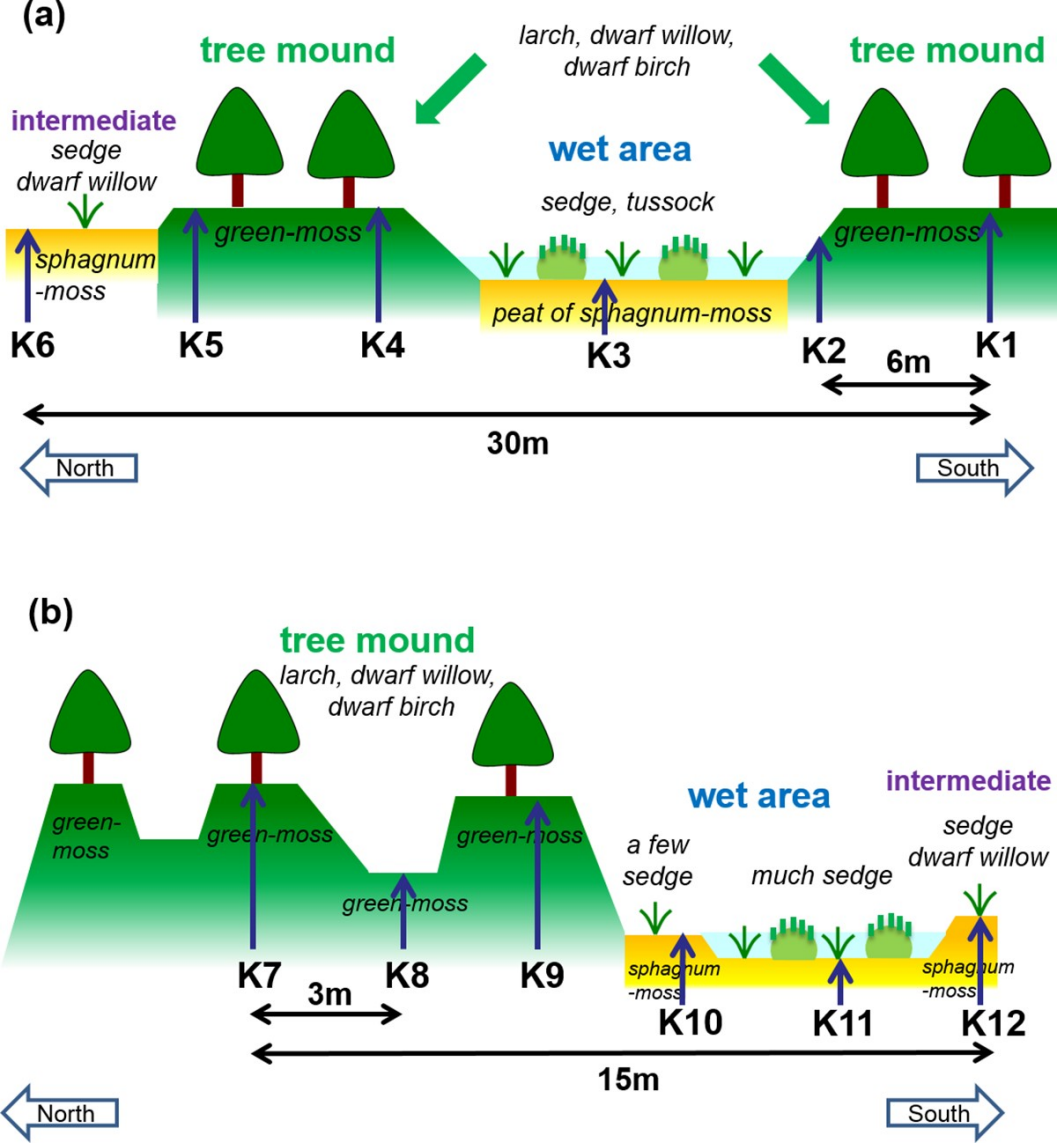
Vegetation and microtopography at the transect sampling points. (a) K1–K6 and (b) K7–K12.

Fig 4 illustrates the microtopography and vegetation composition along the transect sampling points. The understory vegetation of the tree mounds was mainly green-moss, larch, and shrubs (dwarf willow and dwarf birch). That of the wet area was mainly sedge and sphagnum-moss. The average values for the thaw depth, organic layer depth, GWC of the frozen layer, isotopic parameters, relative elevation, and soil density for the samples obtained from the tree mounds and wet areas are given in Table 2. The data obtained from the intermediate area are included with the data from the wet areas.

#### Surface organic layer

**Fig 5:**
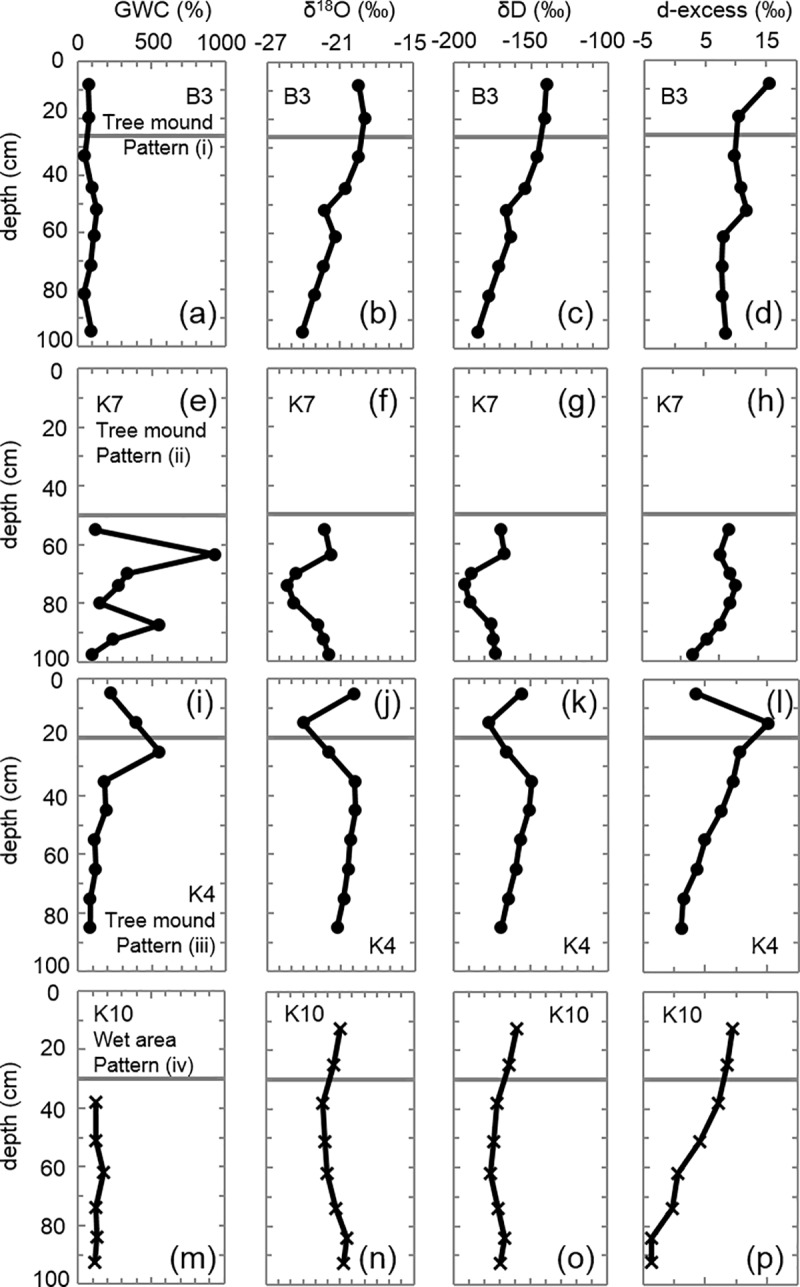
Vertical profiles of the gravimetric water content (GWC) and isotopic compositions of boreholes. (a) GWC, (b) δ^18^O, (c) δD, and (d) d-excess values of borehole B3. (e)–(p) data for K7, K4, and K10. The horizontal line in each figure represents the frozen table, and the vegetation and microtopography (tree mounds or wet areas) and patterns (i–iv) described in the Results section are also shown.

A surface organic layer was observed for all samples (Fig 5 and S1–S4 Figs). The average depths in the tree mounds and wet areas were 31.6±24.0 cm and 26.9±8.6 cm, respectively, for all sites. No statistical difference was observed (Table 2).

#### Thaw depth

Thaw depth of the wet areas (28.6±5.0 cm) tended to be greater than that of the tree mounds (18.8±2.9 cm) at site K (Table 2). A similar tendency was observed at sites A, B, and V. The mean thaw depths of the tree mounds and wet areas were 19.9±3.3 cm and 30.3±6.7 cm, respectively, for all observation sites (Table 2). The soil moisture of the thaw layer in wet areas (around 50% in volumetric water content) was generally higher than that in tree mounds (around 20% or less in volumetric water content) [64–66].

Thaw depth usually reaches a maximum during the first half of September, and the maximum depth is considered to be more than 50 cm based on the data of the seasonal variation in ground temperature (S5 and S6 Figs). Although it appears that the observed ground temperature at 75 cm depth slightly exceeded 0°C, the ground temperature may be artificially high owing to soil disturbance by probe installation.

#### Water and ice contents

The averaged GWCs of the frozen cores from the tree mounds and wet areas were 167.3±71.1% and 92.6±20.0%, respectively, for all sites. Those at site K were 206.3±58.9% and 100.6±19.5%, respectively (Table 2). There were significant differences between the vegetation types at all sites (p < 0.01) and at site K (p < 0.01).

Most of the frozen cores contained excess ice including numerous ice lenses of various thicknesses. Layers with a high ice content of more than 200% were only observed in the tree mounds of site K and other sites, especially at the top of the frozen layer and at the around 60 cm depth or deeper (Fig 5 and S1–S4 Figs). Ice lenses and an ice-rich layer were frequently observed in the frozen layer of the tree mounds but were not common in the wet areas. Consequently, the ice content of the tree mounds often varied with depth, whereas the wet areas showed relatively little variation in the ice content.

#### Stable isotopic composition of water

The average δ^18^O values of ice for the frozen layer in the tree mounds and wet areas were −21.9±0.8‰ and −21.6±0.3‰, respectively, for all sites. No significant difference was observed (Table 2). Those for site K were −21.9±1.0‰ and −21.5±0.2‰, respectively, and no significant difference was observed.

The average d-excess values for the frozen layer in the tree mounds and wet areas were 8.1±1.9‰ and 5.4±3.4‰, respectively, for all sites. Those for site K were 7.2±1.8‰ and 3.6±2.5‰, respectively. Significant differences were observed in the data at all sites, and also in that at site K (Table 2) using the statistical test. However, since the standard deviation of the average d-excess values were less than the precision of d-excess, there may be differences but not significant differences.

### Vertical profiles of the water content, water isotopic ratio, and d-excess

As described above, the tree mounds and wet areas were distinguished by the GWC. We used the δ^18^O and δD values to identify four characteristic vertical profiles. Fig 5 shows the examples of (i) B3, (ii) K7, (iii) K4, and (iv) K10, and the characteristics of their patterns are described below:

- The delta values decreased as the depth of the frozen layer increased.
- The water isotopic ratios showed a different trend in ice-rich layers relative to other layers.
- Low delta and high d-excess values were observed at the bottom of the thaw layer and top of the frozen layer.
- The delta values had a narrow fluctuation range.

#### Pattern (i)

The B3 sample showed a gradual decrease in the delta values from the surface of the frozen layer (33 cm depth) to the bottom of the core (95 cm depth), as shown in Fig 5. δ^18^O decreased from −19.5‰ to −24.1‰ (Fig 5B), and δD decreased from −146‰ to −185‰ (Fig 5C). This pattern was observed in most samples (B1, B2, B3, B4, K1, K2, K4, K5, K6, V1, V2, and A1) except for some samples taken from the wet areas (Table 1).

#### Pattern (ii)

Sample K7 (Fig 5E) showed a high ice content (more than 200%) because of the many ice-rich layers (S7 Fig). δ^18^O and δD decreased (from −25.4‰ to −24.7‰ and from −193‰ to −188‰, respectively) at a depth of approximately 70–80 cm compared to in the adjacent upper and lower ice-rich layers (Fig 5D and 5G). Ice-rich layers were also observed in samples K1, K8, and K9, and their delta values showed different trends relative to other layers (S1 and S2 Figs). All samples categorized in pattern (ii) were observed in the tree mounds (Table 1).

#### Pattern (iii)

The thaw layer at K4 showed a higher water content and d-excess (388.6% and 15‰, respectively) at a depth of 15 cm than at 5 cm (220.0% and 4‰, respectively) (Fig 5I and 5L). On the other hand, the delta values at the 15 cm depth were clearly lower (δ^18^O = −24.1‰, δD = −177‰) than those of the other layers (Fig 5J and 5k). The surface frozen layer in sample K4, which had large amounts of pure ice (S7 Fig), had a higher ice content (550.6%) than the below layer (Fig 5I). The delta values of the layer (δ^18^O = −22.0‰, δD = −166‰) were lower than those of the layer below (Fig 5J and 5K), but d-excess (11‰) was higher (Fig 5L). We found this pattern in a number of 2011 site K cores obtained from tree mounds that had cracks on the surface (Table 1).

#### Pattern (iv)

The ice content of the frozen layer at K10, which was obtained from a wet area (Table 1), was low at approximately 120%, and the variation range was narrow (Fig 5M). Furthermore, there was no ice-rich layer (S7 Fig). The variations in the delta values in the profiles were also narrow, and there was no vertical trend (Fig 5N and 5O). The same trend was observed at K3, K10, K11, K12, V4, and A2, which were in the wet and intermediate areas (Table 1).

### Isotopic composition of ground ice and water

**Fig 6:**
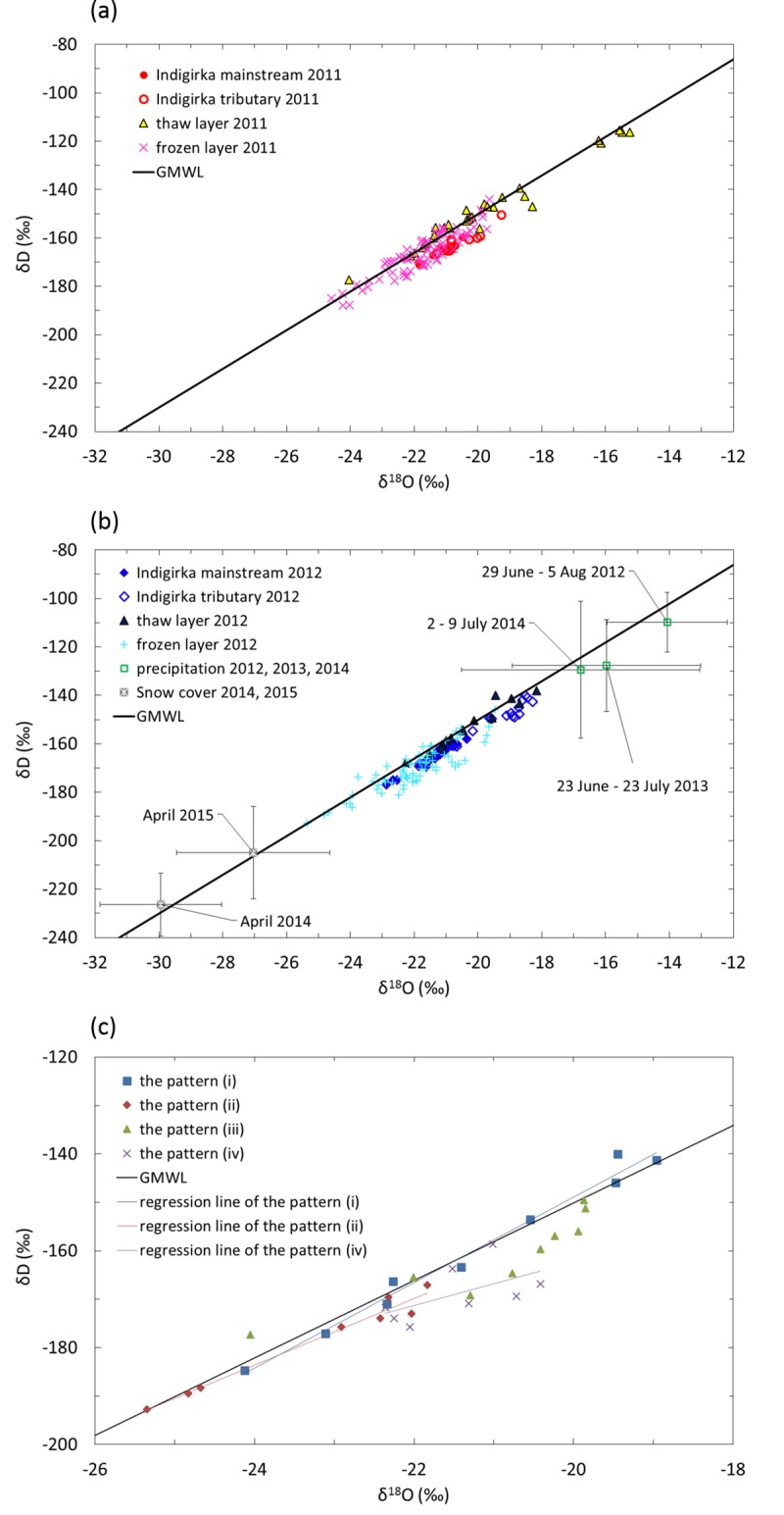
δD–δ^18^O plot for water, ice, and snow samples with the Global Meteoric Water Line (GMWL; δD = 8 × δ^18^O + 10). (a) The δD–δ^18^O plot was obtained for water from the Indigirka mainstream and tributaries, water and ice in the thaw and frozen layers during the summer sampling period in 2011 and (b) similar data observed in 2012. The isotopic compositions of precipitation were observed in 2012, 2013, and 2014. The snow cover was obtained in April 2014 and April 2015. The isotopic compositions of the precipitation and snow cover are shown with the SD. (c) The δD–δ^18^O data of B3, K7, K4, and K10 are plotted as profile patterns (i), (ii), (iii), and (iv). The regression equations of patterns (i), (ii), and (iv) are δD = 8.8 × δ^18^O + 27, δD = 6.9 × δ^18^O – 19, and δD = 4.5 × δ^18^O − 73, respectively.

Fig 6 shows the δD–δ^18^O plot of river water from the Indigirka mainstream and tributaries, water in the thaw layer, ice in the frozen layer, precipitation, and snow cover obtained during the sampling periods. Precipitation data for summers in 2013 and 2014 and snow data obtained in 2014 and 2015 April are also shown. The δD and δ^18^O values of the Indigirka mainstream water ranged from −177‰ to −155‰ and from −22.9‰ to −20.3‰, respectively. The δD and δ^18^O values of the Indigirka tributaries water ranged from −171‰ to −140‰ and from −21.8‰ to −18.3‰, respectively, which are generally higher than the mainstream values. All of the river data slightly deviated below the Global Meteoric Water Line (GMWL) with d-excess of 0‰–8‰, which seems to be the mixing of summer precipitation with low d-excess and evaporated surface water.

The δD and δ^18^O values of the soil water in the thaw layer in 2011 were from −177‰ to −115‰ and from −24.1‰ to −15.3‰, respectively, and those in 2012 were from −168‰ to −138‰ and from −22.3‰ to −18.2‰, respectively (S1 Table). The data in 2011 except for some samples with low delta values (δD and δ^18^O were −177‰ and −24.1‰, respectively) and several high delta values (δD and δ^18^O were from −121‰ to −116‰ and from −16.2‰ to −15.3‰, respectively) were in a comparable range with the data in 2012, which were similar to the delta values in the Indigirka tributaries (Fig 6A and 6B). The δD and δ^18^O values of ice in the frozen layer were lower than those of the water samples from the river and thaw layers with ranges from −193‰ to −144‰ and from −25.4‰ to −19.5‰, respectively. Most of the data of the thaw and frozen layers were plotted on or slightly below the GMWL.

The precipitation mostly had higher average δD and δ^18^O values with the standard deviation (SD) during the summer sampling periods (June–August) in 2012 (−110±12‰ and −14.1±1.9‰, respectively), 2013 (−128±19‰ and −16.0±3.0‰, respectively) and 2014 (−129±28‰ and −16.8±3.7‰, respectively) than those of other samples from the river and water and ice in the soil. The soil water in the thaw layer had very high delta values (Fig 6A) close to the summer precipitation (Fig 6B).

The average δD, δ^18^O, and d-excess values with SD of the snow cover observed in 2014 (−226±13‰, −29.9±1.9‰ and 13±3‰, respectively) and 2015 (−205±19‰, −27.0±2.4‰ and 11±6‰, respectively) clearly had the lowest delta values.

## Discussion

### Relationship between the ground ice and the vegetation and microtopography

As described in the Results section, the tree mounds showed higher GWC in the frozen layer, shallower thaw depth, and higher relative elevation than the wet areas (Table 2). Ice-rich layers were only observed in the frozen layer of the tree mounds. Such data suggest that the presence of ground ice contributes to microtopographic features; namely, the higher elevation of the ground surface is due to frost heave [26].

On the other hand, the soil moisture in the thaw layer at tree mounds was smaller than that at wet areas [64, 66]. The vegetation of the higher elevation areas (tree mound) was larch trees and shrubs with green-moss and cowberry, whereas that of the lower elevation areas (wet area) was mainly sedges and sphagnum-moss. This vegetation structure and composition may depend on the differences in the microtopographic elevation and thaw depth (i.e., frost table elevation), which control the soil moisture condition [18–20].

Similar results on the vegetation structure and composition corresponding to the microtopography have also been reported for Arctic tundra [15–17]. The surface elevation is also affected by the accumulation of mosses and organic matter from the vegetation. The vegetation and microtopography, which are affected by the ground ice, may interact with each other and form or change landscapes in this region.

There seem to be two prominent types and formation processes of ground ice in tree mounds. One is ice segregation as soil freezes. Massive ice segregation is likely to occur particularly at the transition zone, which is situated at the uppermost layer of permafrost and alternates in status between an active layer and permafrost over periods of less than a decade to multiple centuries [27, 28]. At K7 categorized into pattern (ii), the depth of approximately 60–90 cm showed high ice content, which was situated at the upper part of the frozen layer (Fig 5E and S7 Fig). This ice-rich layer led to the transition zone where ice segregation occurred. As described previously, pattern (ii) was observed in tree mounds. A higher surface elevation due to frost heave and the accumulation of organic matter or natural levees of former river channels [66, 69] caused the low soil moisture condition. The lower thermal conductivity with the low soil moisture may have induced further ice segregation with slow freezing, as explained in the next section.

The second process is ice wedge formation due to the infiltration of snowmelt water in thermal contraction cracks (e.g., [33]), which produces polygon mires [14]. A high ice content of almost pure ice with a thickness of less than 10 cm was observed in the uppermost part (20–30 cm depth) of the frozen layer at K4 (Fig 5I and S7 Fig), which was categorized as pattern (iii). The ice wedges of pattern (iii), which were confirmed by isotopic composition as described in the next section, were also observed in tree mounds. The low soil moisture of tree mounds might help form thermal contraction cracks [26].

An ice-rich layer was not observed in the wet areas, which may have caused the lower elevation compared to tree mounds. Although the observed ice content in the frozen layer was small at the wet areas, the soil moisture was higher than the tree mounds [64, 66]. The thermal conductivity of the surface soil layer in the wet area could be high because of the greater soil moisture [29, 30], which may lead to permafrost thawing in summer and quickly freezing in winter. For the tree mounds, the surface shrubs currently increasing in the Arctic region [8, 10], may inversely disturb permafrost thawing through the shading effect [70]. The low soil moisture content [64, 66], green-moss, and organic matter on the surface of the tree mounds are also important factors which decreased thermal conductivity [29, 30]. Lower thermal conductivity at the tree mounds may reduce permafrost thawing during summer and cause slow freezing in winter. As described above, our data suggest that the vegetation and microtopography also affect the ground ice.

### Formation processes and isotopic compositions of the ground ice

The freezing rate, which depends on the thermal conductivity, is an important factor for ground ice formation in soil. It has been also reported that the minimum ground temperature during a winter at the top of permafrost also affected the formation of ground ice (e.g., [21, 71]). Kokelj and Burn [21] showed a large difference in the minimum ground temperature at the top of permafrost among sites (more than 10°C) at Mackenzie Delta, and this affected the difference in formation of ground ice. In our sites, however, minimum ground temperature was about −14°C in the 2011 and 2012 winter, which was the same between tree mounds and wet areas (S5 and S6 Figs). Therefore, it is considered that there was no difference in the effect of the minimum ground temperature between tree mounds and wet areas for the formation of ground ice in the sites.

The freezing rate also affects isotope fractionation during freezing of water. The ice formation processes of each profile pattern were assessed according to the isotopic composition of the ground ice.

In the frozen cores categorized into pattern (ii), large variations in the delta values were observed for the ice-rich layer with high ice content (Fig 5E–5G); this indicates isotope fractionation during ice formation. Prominent fractionation was observed at the depth of 60–90 cm in the frozen cores. These layers had high ice content, and the δ^18^O values were also higher at the upper and lower parts of the layers (−21.8‰ and −22.9‰) than at the middle parts of the layers (from −25.7‰ to −24.7‰). The slope of the regression line of pattern (ii) in the δD–δ^18^O plot was 6.9 (Fig 6C), which suggested a near equilibrium isotope fractionation during freezing [38–40] (cf. S1 Appendix). The δ^18^O value of ice has been reported to be enriched by 2.8–3.1‰ compared to the source water when frozen under equilibrium conditions [38, 40]. If the initial δ^18^O value of the water was −23.8‰, which was the average δ^18^O at a depth of 60–90 cm, the δ^18^O value of the first ice will be from −21.0‰ to −20.6‰. That of the subsequent ice will gradually decrease and finally be quite lower than the initial value (cf. S1 Appendix). Therefore, it is indicated that the upper part (60 cm depth) and the lower part (90cm depth) were formed at first (higher δ^18^O values), and then the ice of middle part at the depth of 60–90 cm was formed (lower δ^18^O values). Observed δ^18^O values of the first ices were slightly lower than the theoretical values due to thickness of the sampling.

The equilibrium fractionation during freezing occurs when the freezing rate is less than the HDO and H_2_^18^O diffusion rates [39]. The slow freezing may provide sufficient time for unfrozen soil water to slowly migrate to the freezing front. Soil freezing in winter generally starts from the upper side because of the cooling air temperature, and also from the lower side because of the permafrost. Therefore, the unfrozen soil water apparently migrated to both freezing fronts and formed thick ice lenses, so higher ice contents were observed at the depths of 60 and 90 cm (917% and 542%, respectively), and lower ice content was observed at the middle part between them (149%) (Fig 5E).

The delta values tended to decrease from the surface soil to the deeper frozen layer in most of the samples categorized in pattern (i), as shown in Fig 5B and 5C. These trends suggest Rayleigh-type fractionation of the stable water isotopes during freezing (e.g., [39, 72–74]) (cf. S1 Appendix). Additionally, discontinuities in the profiles of the delta values and d-excess were observed at a depth of 52–61 cm (Fig 5B–5D), which indicates thaw unconformity. This depth appears to be the maximum depth of the active layer (S5 and S6 Figs). The decrease in the delta values may have been caused by isotopic fractionation during freezing and developed above and below the layers of the thaw unconformity separately.

Pattern (iii) showed a high ice content at the uppermost frozen layer (551% in Fig 5I) with δ^18^O and δD values of −22.0‰ and −166‰, respectively. On the other hand, the delta values of the lowermost thaw layer were low (δ^18^O of −24.1‰ and δD of −177‰), and the d-excess value was high at 15‰ (Fig 5J–5L). This suggests a large contribution from snowmelt water. As indicated in Fig 6B and S1 Table, snow cover showed low δD and δ^18^O values with high d-excess. The δD and δ^18^O values (and d-excess) observed in 2014 were −226±13‰ and −29.9±1.9‰ (and 13±3‰), respectively, and those in 2015 were −205±19‰ and −27.0±2.4‰ (and 11±6‰), respectively. The snowmelt water mixed with soil moisture from the previous summer may have been source water of the ice. This water infiltrated from the surface to the frozen layer and then froze as an ice wedge because the air temperature was greater than 0°C and the frozen ground was colder than 0°C.

The difference between the isotopic values of the lowermost thaw layer and ice wedge (approximately 2‰ for δ^18^O and 4‰ for d-excess) in pattern (iii) may have been due to isotope fractionation. We sampled the ice wedge separately from just below the frozen layer as seen in S7 Fig. Therefore, we obtained the δ^18^O of total ice of the ice wedge, and compared with soil moisture in the thaw layer. This difference was slightly lower than the theoretical values (2.8‰ for δ^18^O) if we used the fractionation factor by Suzuoki and Kimura [38]. This observational result showed that fractionation factor was slightly lower, and freezing rate was slightly larger than the diffusion rates, leading to the fluctuation of the delta values decreased [38, 39]. Although the freezing rate does not affect the ice wedge formation directly, the location where the ice wedge formed was at the sites with slow freezing rates, such as tree mounds with low soil moisture (and thus low thermal conductivity). This environment may be an important factor to suppress the thawing and growth of ice wedges.

The frozen layer in pattern (iv) had alternating layers of fine ice lenses and clays with low ice content of approximately 130% (Fig 5M and S7 Fig). In other words, there was no ice-rich layer. The d-excess value seemed to be lower for the frozen layers of the wet areas than for the tree mounds (Table 2), and the slope of the regression line between δD and δ^18^O of pattern (iv) was 4.5 (Fig 6C), which is considerably less than that of the other patterns. Such data suggest that the ice freezing occurred rapidly (i.e., under relatively non-equilibrium conditions) in the layer [38, 39]. This rapid freezing, especially when the freezing rate was far greater than the HDO and H_2_^18^O diffusion rate, does not allow sufficient time for ice segregation and isotopic fractionation to occur. Accordingly, the ice content stayed low (i.e., an ice-rich layer did not form), and the delta values were almost constant for pattern (iv). The rapid freezing may have been due to the relatively high thermal conductivity.

The different thermal conductivity in the surface soil coexisting in the same area (i.e., the different freezing rates between tree mounds and wet areas) may depend on the vegetation and microtopography. The main controlling factors of the thermal conductivity are the soil moisture [29, 30], snow cover [31], and organic soil [30]. Compared to the wet areas, the tree mounds had lower soil moisture [64–66], and thicker snow cover (e.g., [75, 76]). There was no difference in the depth of organic layer (Table 2), although more leaves fell down from the trees. Therefore, the thermal conductivity in winter (i.e., the freezing rate) of the tree mounds was likely lower than that of the wet areas.

There was no difference in average water isotope values of frozen layers between tree mounds and wet areas (Table 2), and these isotope values (δD-δ^18^O) were plotted between summer precipitation and snow cover (Fig 6). Therefore, it is clear that the mixture of summer precipitation and snow cover infiltrated the soil, and served as a source of the ground ice. However, it is unclear when this water was infiltrated and formed ices, because currently the tree mounds with dryer surface soils show more ground ice in frozen layers than in wet areas.

During *in situ* freezing of soil, the freezing rate might be affected by such as the presence of unfrozen water in the soil, differences in soil particles, and heterogeneous soil temperature. They are further complicated by the combination of these factors, and the freezing is not clearly differentiated between the equilibrium or non-equilibrium conditions. There are many unknown points about the *in situ* freezing of soil, which requires further investigation.

The freezing rate (i.e., the thermal conductivity in the surface soil) affects the formation of the ground ice, therefore, the future changes in the snow cover, soil moisture, and organic layer predicted in models (e.g., [77–81]) can impact the freezing environment of the ground ice. In addition, a warming trend has been reported for the permafrost in northeastern Siberia [82, 83], and numerical models have calculated significant degradation of the permafrost during the 21st century [84, 85]. This suggests that a difference in the relative elevation (i.e., microtopography) may be reduced by a loss of ground ice thickness, which can have a significant impact on the vegetation structure and composition in northeastern Siberia. To reduce the uncertainty over the future conditions of the ground ice, the response of the soil environment (i.e., soil moisture, organic soil) to climate change needs to be further investigated.

## Conclusions

In the current study, we investigated the spatial distribution of the ice content and the stable isotopic composition of ice and water in the near-surface permafrost to a depth of 1 m in relation to the vegetation and microtopography of the Taiga–Tundra boundary in the Indigirka River lowlands of northeastern Siberia. An ice-rich layer was observed in the tree mounds (microtopographically high elevation areas with growing larch trees), which had a significantly higher GWC than in the wet areas (lower elevation with wetland vegetation). This suggests that the relatively high microtopography of the land surface due to frost heave strongly affects vegetation composition. The water isotopic composition of the ground ice indicated that ice segregation occurred under close to equilibrium condition (slow freezing) in the tree mounds, whereas the near-surface ground in the wet areas froze under relatively non-equilibrium conditions (rapid freezing). Our results suggest that the lower thermal conductivity in the surface soil (i.e., the slower freezing rate) of the tree mounds compared with the wet areas was due to lower soil moisture and thicker snow cover. Therefore, future possible changes in snow cover, soil moisture, and organic soil, which also depend on the vegetation and microtopography, could have significant impacts on the freezing environment of the ground ice at the Taiga–Tundra boundary of northeastern Siberia in the Arctic region. These changes in the freezing environment may then in turn affect the vegetation through changes in the microtopography of the ground surface.

The results of this study should greatly contribute to research on permafrost degradation and associated vegetation and microtopography changes at the Taiga–Tundra boundary in northeastern Siberia. Future work will involve collecting data from other Taiga and Taiga–Tundra boundary areas and conducting experimental studies on soil freezing. Further investigations on vegetation and microtopography changes are necessary to predict permafrost degradation due to climate change.

## Supporting information

S1 Fig Vertical profiles of the gravimetric water content (GWC) and isotopic compositions of boreholes obtained at site K in 2011. The upper panels show the GWC and the lower panels show δ^18^O, δD, and d-excess values for the observed data at K6, K5, K4, K3, K2, and K1. The horizontal line in each figure represents the frozen table.
(PDF)

S2 Fig Vertical profiles of the gravimetric water content (GWC) and isotopic compositions of boreholes obtained at site K in 2012. The data are similar to those in S1 Fig but for K7, K8, K9, K10, K11, and K12.
(PDF)

S3 Fig Vertical profiles of the gravimetric water content (GWC) and isotopic compositions of boreholes obtained at sites B, V, and A in 2011. The data are similar to those in S1 Fig but for B1, B2, V1, V2, A1, and A2.
(PDF)

S4 Fig Vertical profiles of the gravimetric water content (GWC) and isotopic compositions of boreholes obtained at sites B, V, and A in 2012. The data are similar to those in S1 Fig but for B3, B4, V3, and V4, respectively.
(PDF)

S5 Fig Seasonal variation in ground temperature from August 2010 to July 2012 at site K. The seasonal variations in ground temperature from the surface to 100 cm depth at (a) tree mound and (b) wet area. The ground temperature was measured with thermistor sensors (TMC-HD; Onset Computer Co.) and recorded by data loggers (HOBO U12-006; Onset Computer Co.). Gaps in the time series or absence of the data shows missing data. The data from August 2010 to July 2011 were also reported by Iwahana et al. [50].
(PDF)

S6 Fig Seasonal variation in ground temperature from August 2010 to July 2012 at sites B and V. The seasonal variations in ground temperature from the surface to 100 cm depth at (a) tree mound of site B, (b) tree mound of site V, and (c) wet area of site V. The ground temperature was measured with thermistor sensors (TMC-HD; Onset Computer Co.) and recorded by data loggers (HOBO U12-006; Onset Computer Co.). Gaps in the time series or absence of the data shows missing data. The data from August 2010 to July 2011 were also reported by Iwahana et al. [50].
(PDF)

S7 Fig Cryostructures of core samples B3, K7, K4, and K10. Examples of cryostratigraphic features in frozen layer of the study sites were reported in Iwahana et al. [50].
(PDF)

S1 Table Summary of the δD, δ^18^O, and d-excess results for the water, ice and snow samples. The maximum, minimum, and average values with the SD and the number (n) of samples observed for each year are presented.
(PDF)

S1 File Geological and isotopic data. (XLSX)

S2 File Ground temperature at site K. (XLSX)

S3 File Ground temperature at site B. (XLSX)

S4 File Ground temperature at site V. (XLSX)

S5 File Permission from Polar Science. (JPG)

S6 File Permission from ESRI. (PDF)

S1 Appendix The equations of Rayleigh-type isotope fractionation and the variations in isotopic compositions of ice during equilibrium freezing of water. (PDF)
