# Supplementary figures and images for: Isotopic compositions of ground ice in near-surface permafrost in relation to vegetation and microtopography at the Taiga–Tundra boundary in the Indigirka River lowlands, northeastern Siberia
Source: PLoS One. 2019 Oct 10;14(10):e0223720. doi: 10.1371/journal.pone.0223720 (PMC6786563; doi:10.1371/journal.pone.0223720)

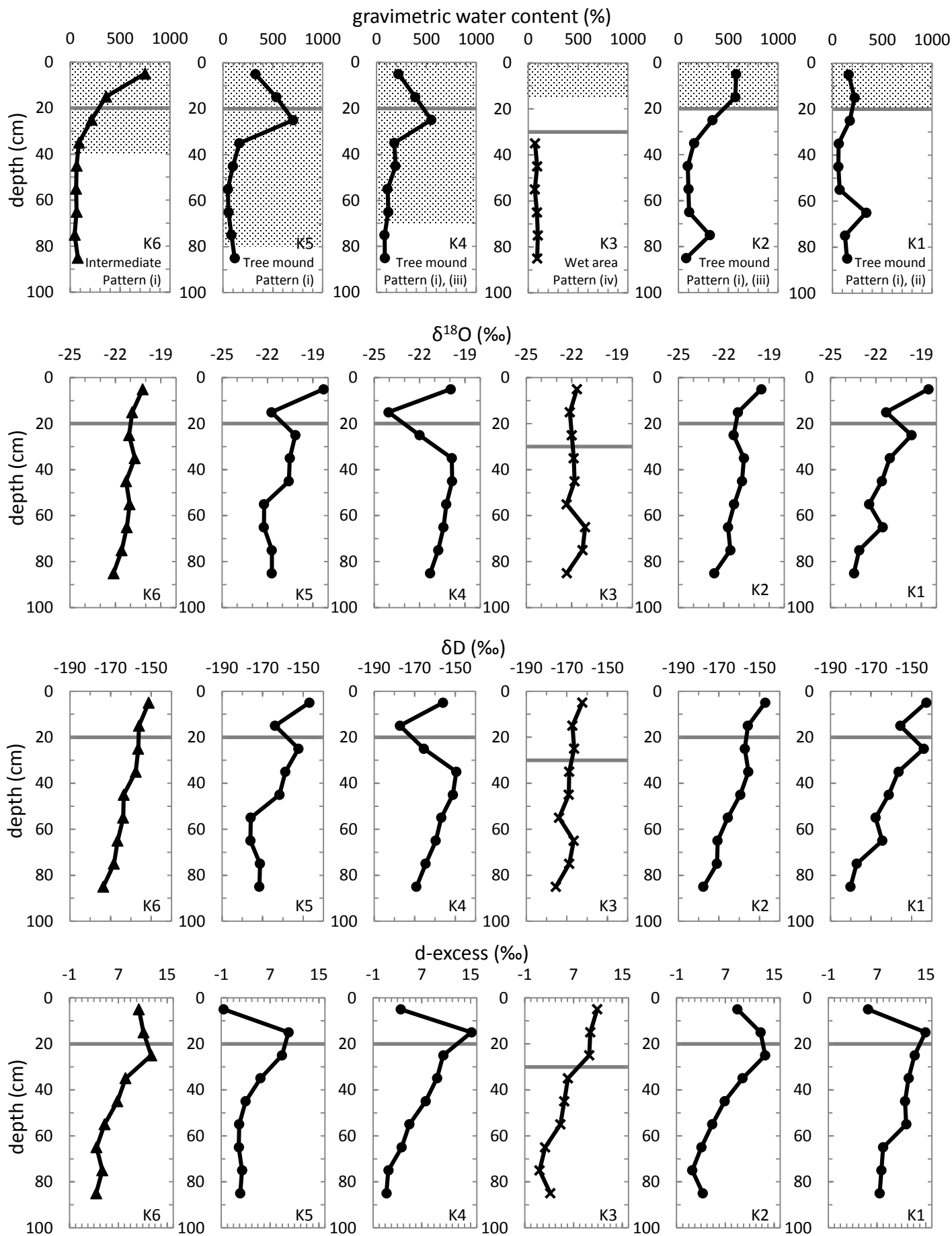

Supplement: S1 Fig — The upper panels show the GWC and the lower panels show δ18O, δD, and d-excess values for the observed data at K6, K5, K4, K3, K2, and K1. The horizontal line in each figure represents the frozen table. (PDF) [file pone.0223720.s001.pdf]

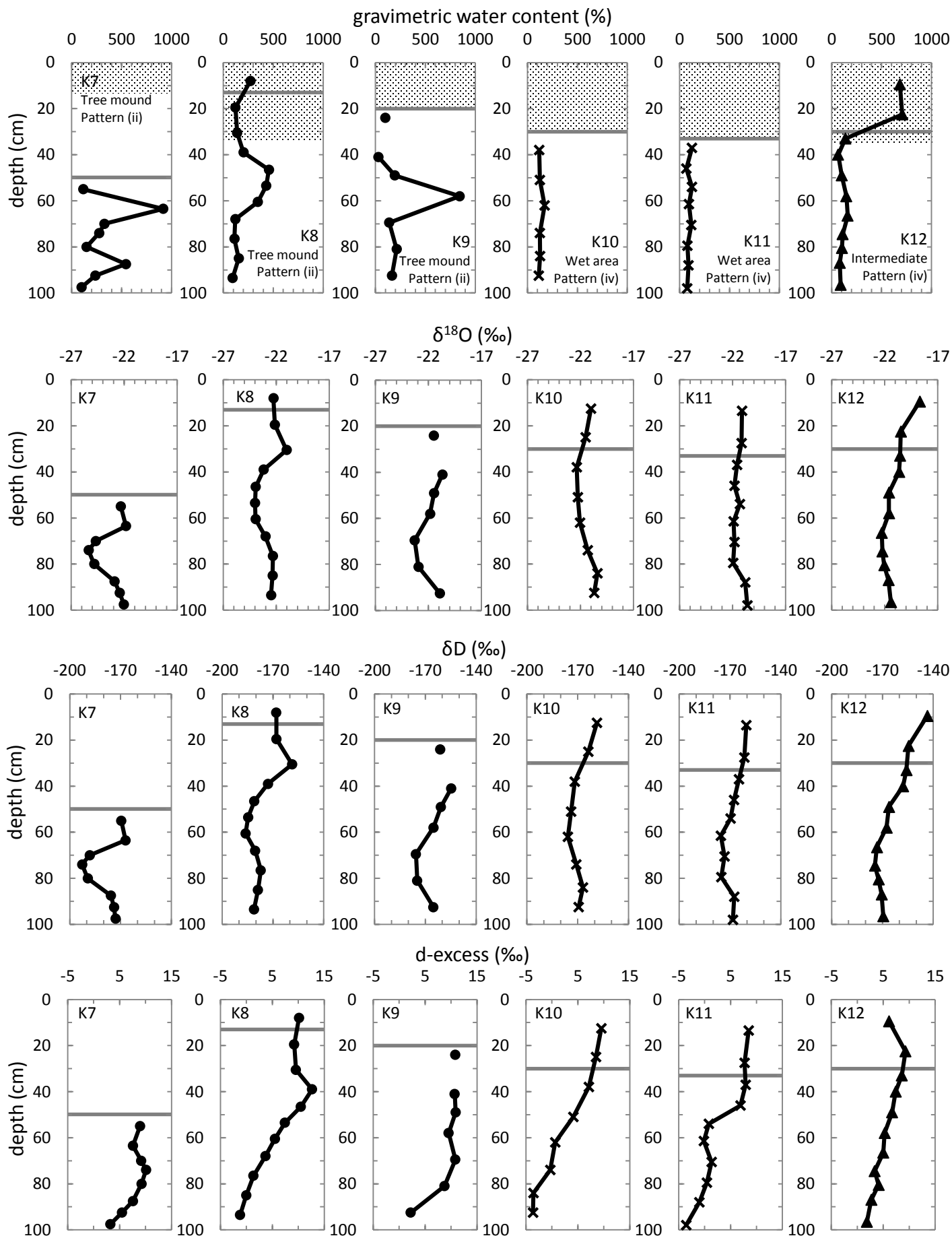

Supplement: S2 Fig — The data are similar to those in S1 Fig but for K7, K8, K9, K10, K11, and K12. (PDF) [file pone.0223720.s002.pdf]

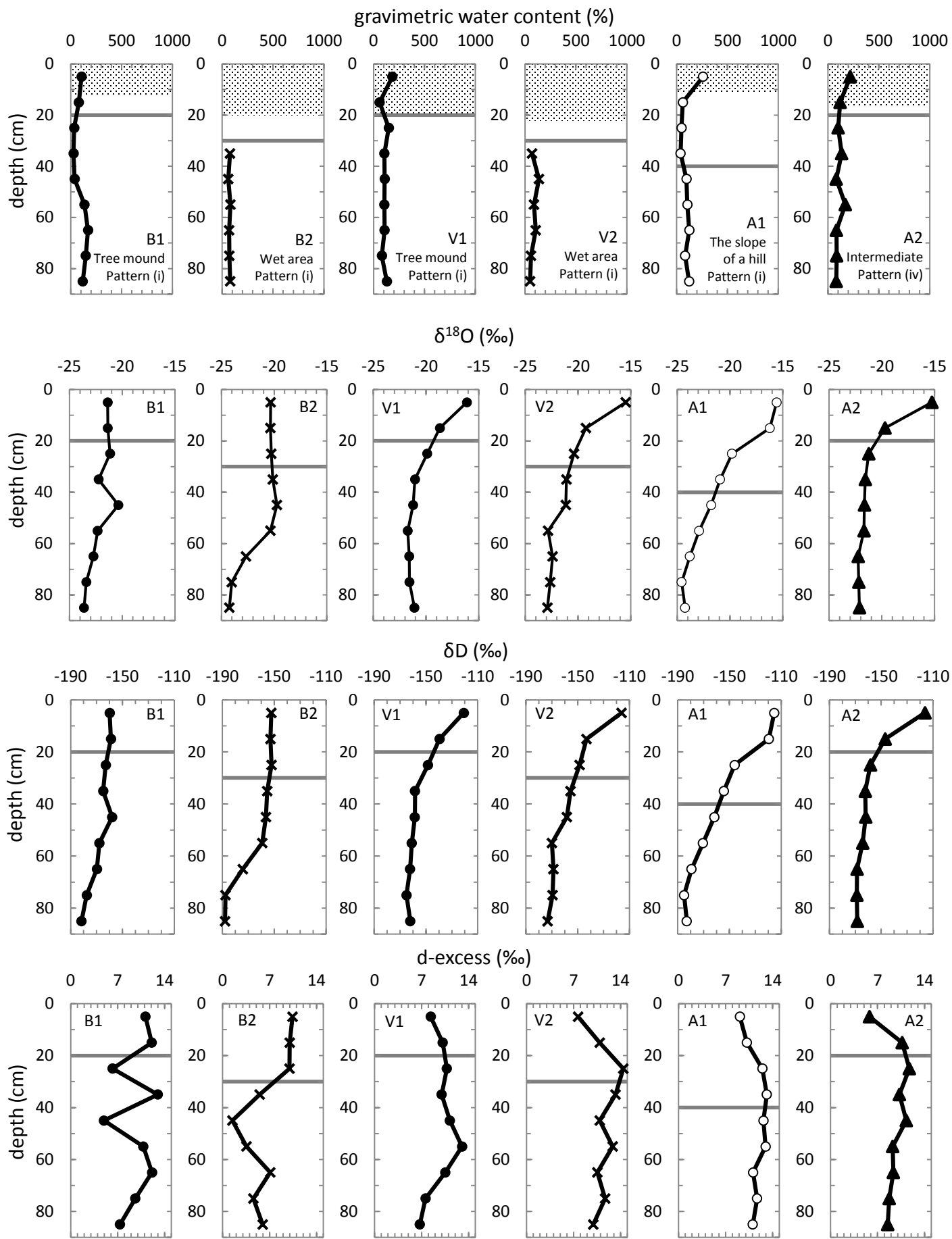

S3 Fig

Supplement: S3 Fig — The data are similar to those in S1 Fig but for B1, B2, V1, V2, A1, and A2. (PDF) [file pone.0223720.s003.pdf]

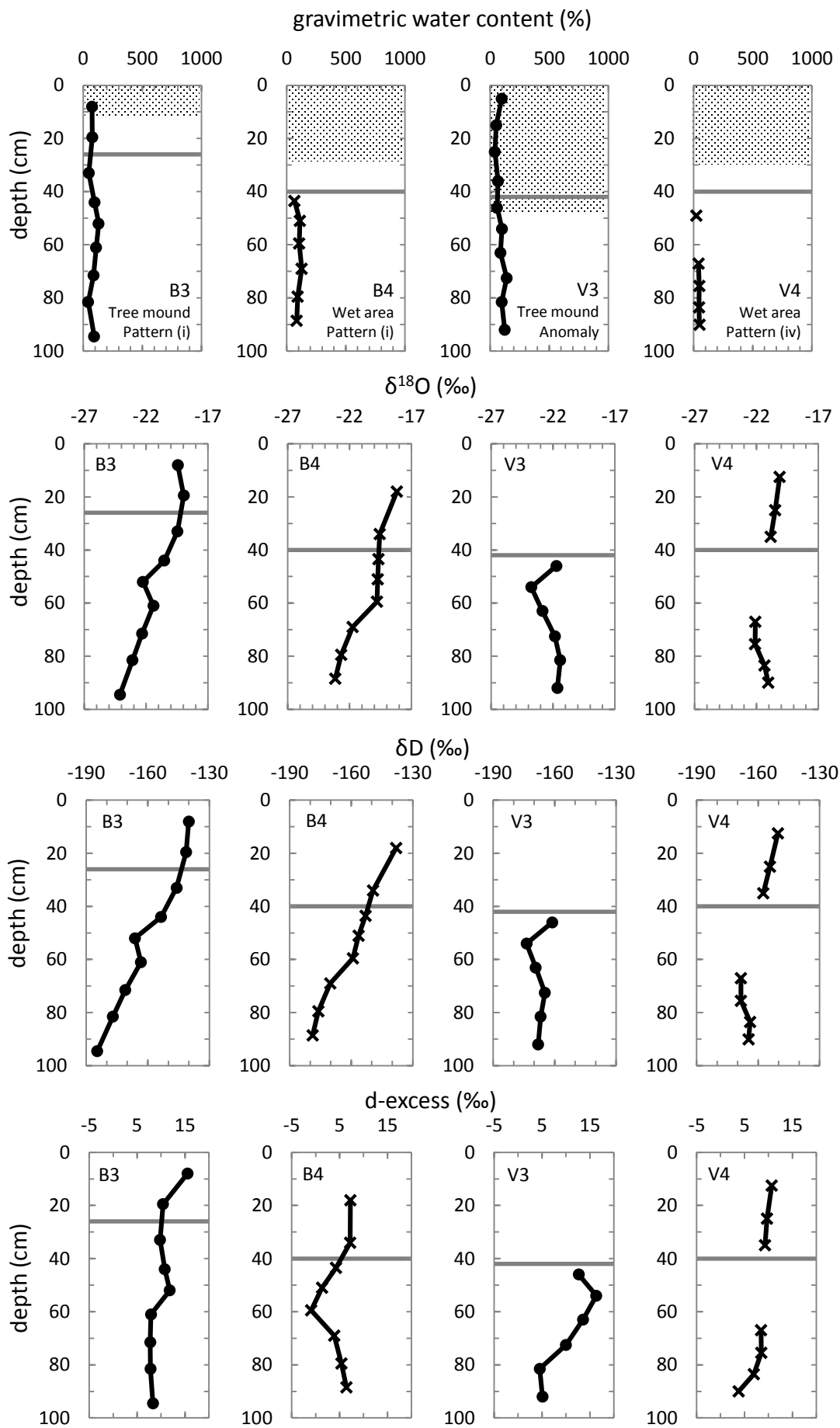

Supplement: S4 Fig — The data are similar to those in S1 Fig but for B3, B4, V3, and V4, respectively. (PDF) [file pone.0223720.s004.pdf]

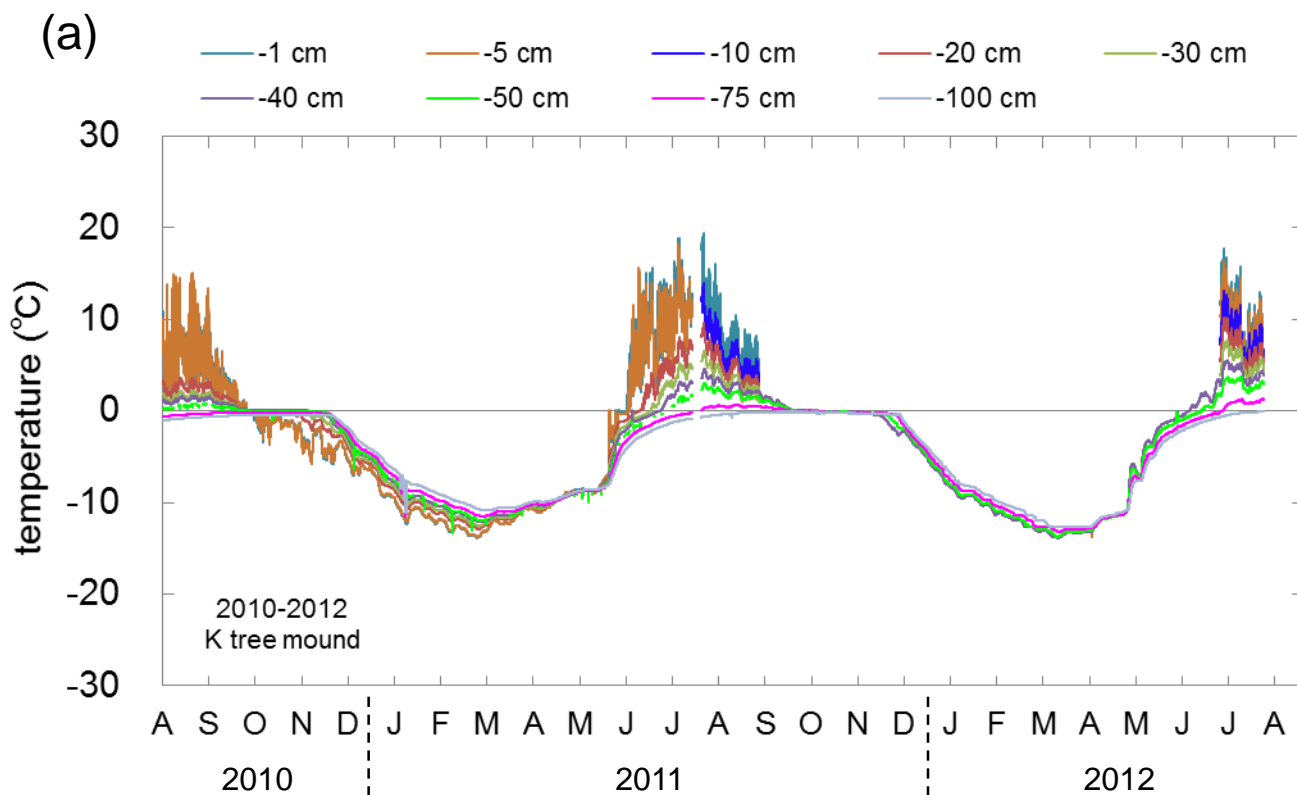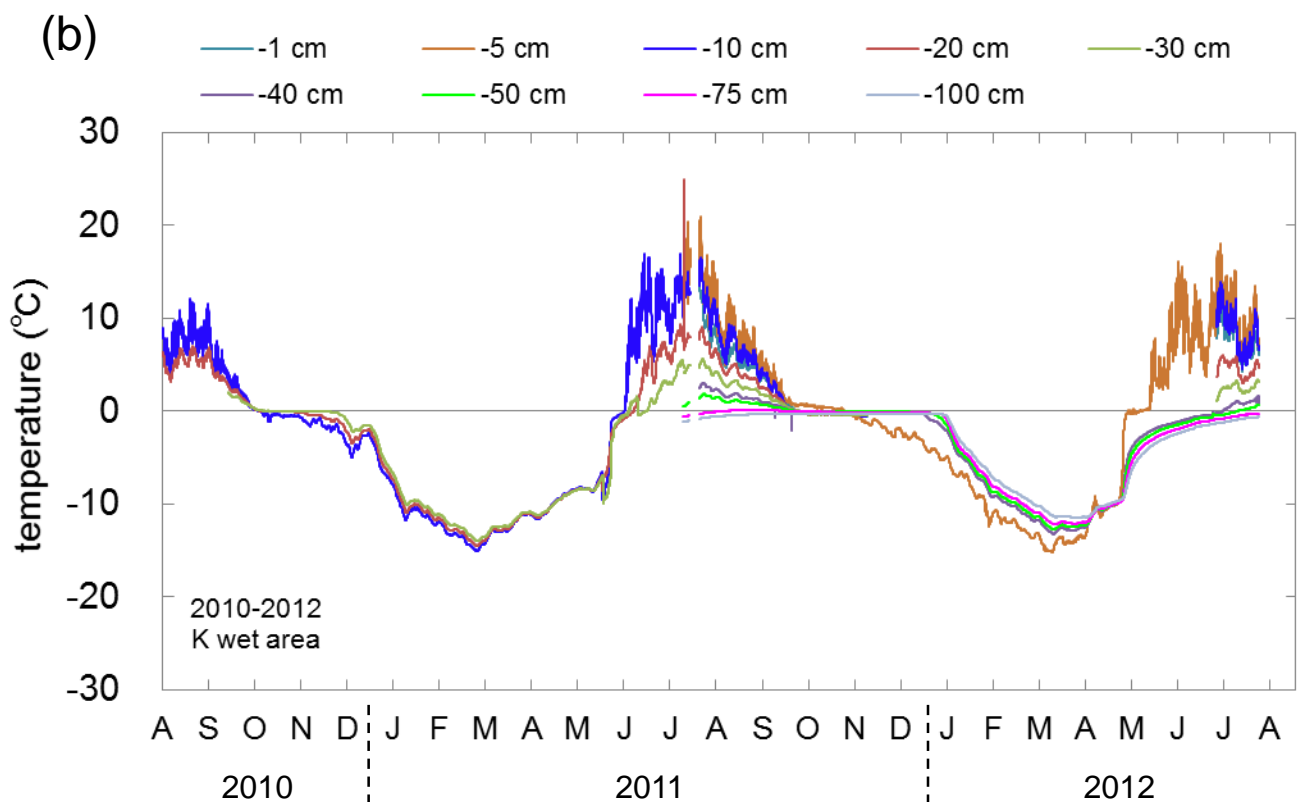

S5 Fig

Supplement: S5 Fig — The seasonal variations in ground temperature from the surface to 100 cm depth at (a) tree mound and (b) wet area. The ground temperature was measured with thermistor sensors (TMC-HD; Onset Computer Co.) and recorded by data loggers (HOBO U12-006; Onset Computer Co.). Gaps in the time series or absence of the data shows missing data. The data from August 2010 to July 2011 were also reported by Iwahana et al. [50]. (PDF) [file pone.0223720.s005.pdf]

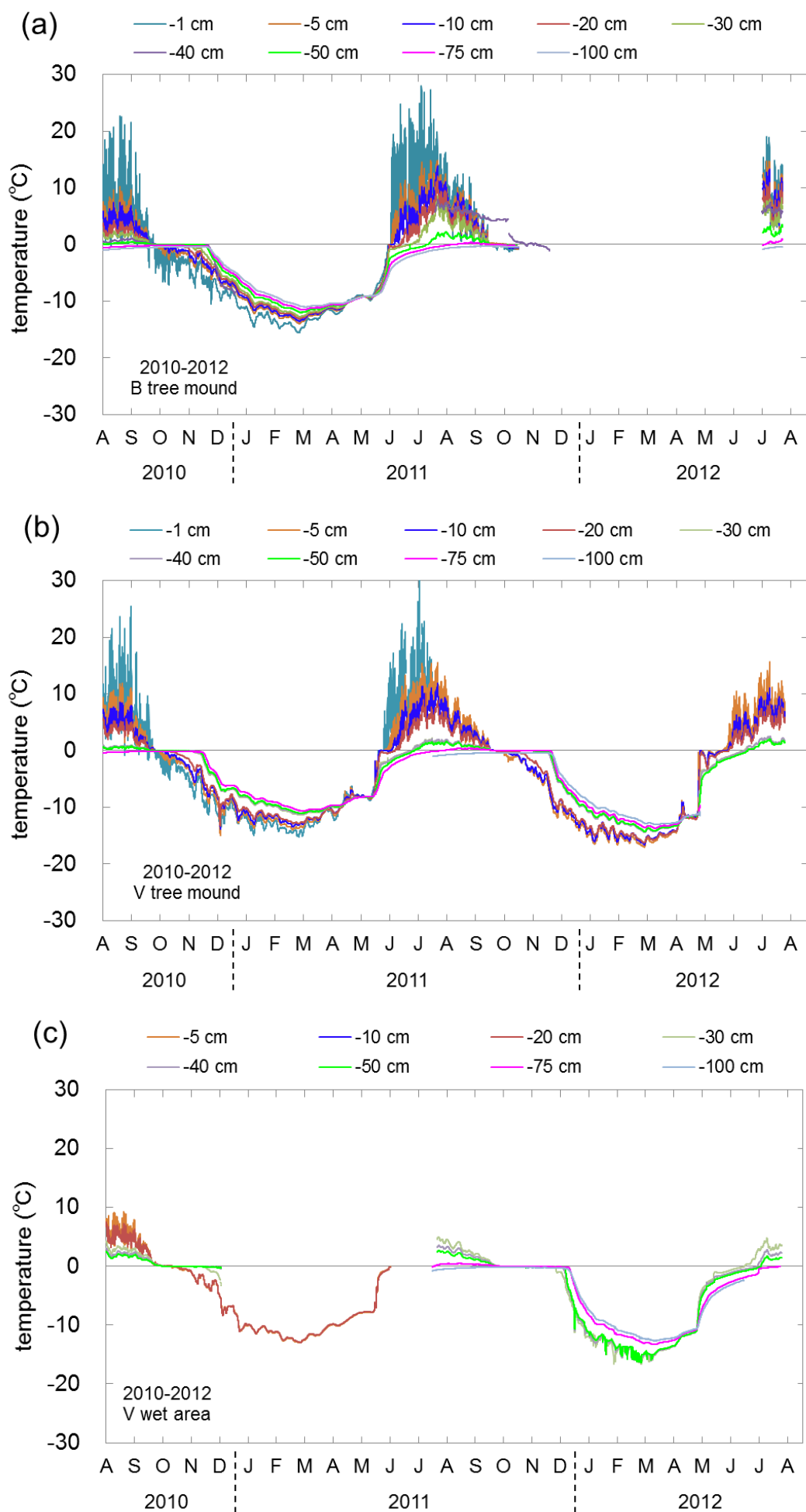

S6 Fig

Supplement: S6 Fig — The seasonal variations in ground temperature from the surface to 100 cm depth at (a) tree mound of site B, (b) tree mound of site V, and (c) wet area of site V. The ground temperature was measured with thermistor sensors (TMC-HD; Onset Computer Co.) and recorded by data loggers (HOBO U12-006; Onset Computer Co.). Gaps in the time series or absence of the data shows missing data. The data from August 2010 to July 2011 were also reported by Iwahana et al. [50]. (PDF) [file pone.0223720.s006.pdf]

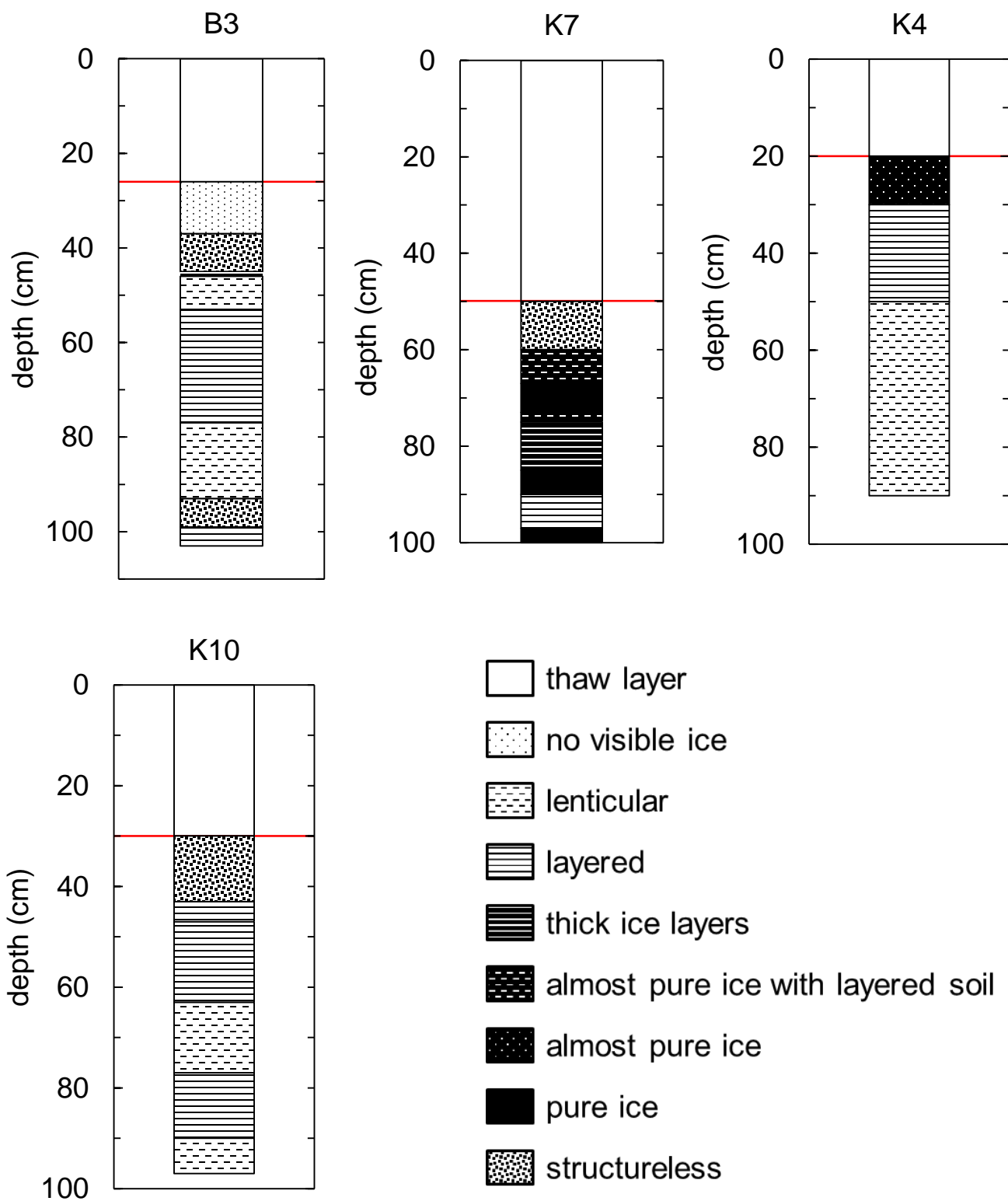

S7 Fig

Supplement: S7 Fig — Examples of cryostratigraphic features in frozen layer of the study sites were reported in Iwahana et al. [50]. (PDF) [file pone.0223720.s007.pdf]

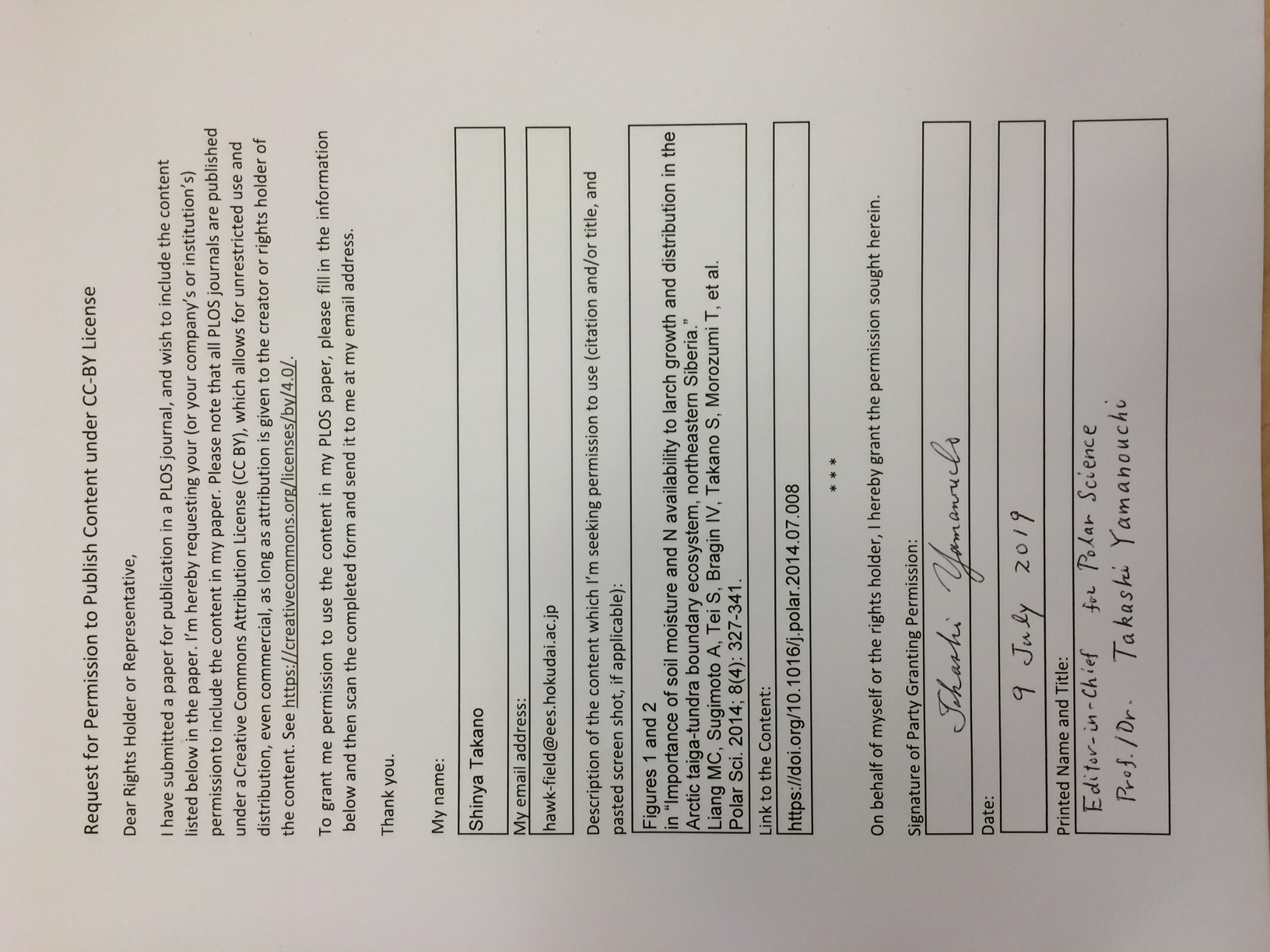

Supplement: S5 File — (JPG) [file pone.0223720.s013.jpg]
