# Supplementary material for: Isotopic compositions of ground ice in near-surface permafrost in relation to vegetation and microtopography at the Taiga–Tundra boundary in the Indigirka River lowlands, northeastern Siberia
Source: PLoS One. 2019 Oct 10;14(10):e0223720. doi: 10.1371/journal.pone.0223720 (PMC6786563; doi:10.1371/journal.pone.0223720)
Supplement: S1 Table — The maximum, minimum, and average values with the SD and the number (n) of samples observed for each year are presented. (PDF) [file pone.0223720.s008.pdf]

S1 Table

	Year	δD (‰)			$\delta^{18}O$ (‰)			d-excess (‰)			n
		Max	Min	Average ± SD	Max	Min	Average ± SD	Max	Min	Average ± SD	
Indigirka mainstream	2011	-160	-170	-164 ± 3	-20.5	-21.9	-21.0 ± 0.4	5	2	4 ± 1	11
	2012	-158	-177	-165 ± 5	-20.3	-22.9	-21.3 ± 0.6	8	3	6 ± 1	29
Indigirka tributary	2011	-150	-171	-163 ± 5	-19.3	-21.8	-20.7 ± 0.6	6	0	3 ± 2	15
	2012	-140	-161	-148 ± 6	-18.3	-20.7	-19.1 ± 0.7	8	2	5 ± 2	14
thaw layer	2011	-115	-177	-148 ± 16	-15.3	-24.1	-19.7 ± 2.2	15	-1	10 ± 3	29
	2012	-138	-168	-153 ± 9	-18.2	-22.3	-20.3 ± 1.2	16	6	9 ± 2	14
frozen layer	2011	-144	-188	-166 ± 9	-19.7	-24.6	-21.7 ± 1.1	14	1	8 ± 4	79
	2012	-146	-193	-170 ± 9	-19.5	-25.4	-22.0 ± 1.2	16	-4	6 ± 4	71
precipitation	2012	-82	-127	-110 ± 12	-10.2	-17.0	-14.1 ± 1.9	10	-3	3 ± 5	12
	2013	-104	-161	-128 ± 19	-12.4	-21.0	-16.0 ± 2.9	8	-17	0 ± 9	9
	2014	-105	-187	-129 ± 28	-13.5	-24.3	-16.8 ± 3.7	8	1	5 ± 2	7
snow cover	2014	-200	-275	-226 ± 13	-25.5	-36.5	-29.9 ± 1.9	20	4	13 ± 3	37
	2015	-172	-258	-205 ± 19	-22.8	-33.4	-27.0 ± 2.4	22	-5	11 ± 6	37
