# Supplementary material for: Isotopic compositions of ground ice in near-surface permafrost in relation to vegetation and microtopography at the Taiga–Tundra boundary in the Indigirka River lowlands, northeastern Siberia
Source: PLoS One. 2019 Oct 10;14(10):e0223720. doi: 10.1371/journal.pone.0223720 (PMC6786563; doi:10.1371/journal.pone.0223720)
Supplement: S1 Appendix — (PDF) [file pone.0223720.s015.pdf]

Rayleigh-type isotope fractionation between water and ice during freezing under isotopically equilibrium conditions can be expressed by the following equations (Lacelle, 2011):

$$R = R_0 f^{(\alpha_{i-w}-1)} + (\alpha_{i-w} - 1)$$

$$\delta_i = \delta_0 + \ln(\alpha_{i-w}) \cdot 1000 \cdot \ln f + \ln(\alpha_{i-w}) \cdot 1000$$

R: the isotope ratio of $^{18}O/^{16}O$ or D/H
R_0: the initial isotope ratio of $^{18}O/^{16}O$ or D/H
$\alpha_{i\text{-}w}$: the equilibrium isotope fractionation factor between water and ice
f: the residual fraction of water during freezing
δ_i: the isotopic composition of ice (formed at that time)
δ_0: the initial isotopic composition of water

The equilibrium isotope fractionation factors between water and ice ($\alpha_{i\text{-}w}$) of $^{18}O/^{16}O$ and D/H are 1.0028 and 1.0206, respectively (Suzuoki and Kimura, 1973). When the initial isotopic compositions of water ($\delta_0{}^{18}O$ and δ_0D) were −20‰ and −150‰, respectively, the isotopic compositions of water ($\delta^{18}O$ and δD) and ice ($\delta_i{}^{18}O$ and δ_iD) were calculated as shown in the following table. The isotopic compositions of ice were plotted in the following figure (δD–$\delta^{18}O$ plot).

			ice (formed at that time)		total ice		water	
initial water	$\alpha_{i\text{-}w}$	*f*	$\delta_i{}^{18}O$ (‰)	δ_iD (‰)	$\delta^{18}O$ (‰)	δD (‰)	$\delta^{18}O$ (‰)	δD (‰)
$\delta_0{}^{18}O$ = -20‰	1.0028	1	-17.2	-130	---	---	-20.0	-150
δ_0D = -150‰	1.0206	0.9	-17.5	-132	-17.4	-133	-20.3	-152
		0.8	-17.8	-134	-17.6	-134	-20.6	-154
		0.7	-18.2	-137	-17.7	-135	-21.0	-156
		0.6	-18.6	-140	-17.9	-137	-21.4	-159
		0.5	-19.1	-144	-18.1	-138	-21.9	-162
		0.4	-19.8	-148	-18.3	-139	-22.5	-166
		0.3	-20.6	-154	-18.6	-141	-23.3	-171
		0.2	-21.7	-162	-18.9	-143	-24.4	-178
		0.1	-23.6	-177	-19.3	-146	-26.3	-189

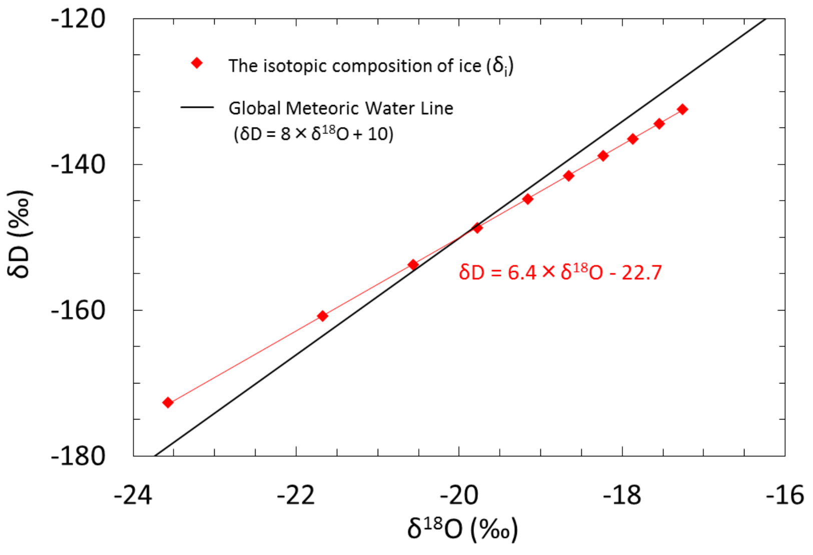

S1 Appendix
